# Induction of NTPDase1/CD39 by Reactive Microglia and Macrophages Is Associated With the Functional State During EAE

**DOI:** 10.3389/fnins.2019.00410

**Published:** 2019-04-26

**Authors:** Marija Jakovljevic, Irena Lavrnja, Iva Bozic, Ana Milosevic, Ivana Bjelobaba, Danijela Savic, Jean Sévigny, Sanja Pekovic, Nadezda Nedeljkovic, Danijela Laketa

**Affiliations:** ^1^Department of Neurobiology, Institute for Biological Research “Siniša Stanković”, University of Belgrade, Belgrade, Serbia; ^2^Département de Microbiologie-Infectiologie et d’Immunologie, Faculté de Médecine, Université Laval, Quebec City, QC, Canada; ^3^Centre de Recherche du CHU de Québec, Université Laval, Quebec City, QC, Canada; ^4^Department for General Physiology and Biophysics, Faculty of Biology, University of Belgrade, Belgrade, Serbia

**Keywords:** NTPDase1/CD39, EAE, ATP, neuroinflammation, microglia/macrophages, astrocytes

## Abstract

Purinergic signaling is critically involved in neuroinflammation associated with multiple sclerosis (MS) and its major inflammatory animal model, experimental autoimmune encephalomyelitis (EAE). Herein, we explored the expression of ectonucleoside triphosphate diphosphohydrolase1 (NTPDase1/CD39) in the spinal cord, at the onset (Eo), peak (Ep), and end (Ee) of EAE. Several-fold increase in mRNA and in NTPDase1 protein levels were observed at Eo and Ep. *In situ* hybridization combined with fluorescent immunohistochemistry showed that reactive microglia and infiltrated mononuclear cells mostly accounted for the observed increase. Colocalization analysis revealed that up to 80% of Iba1 immunoreactivity and ∼50% of CD68 immunoreactivity was colocalized with NTPDase1, while flow cytometric analysis revealed that ∼70% of mononuclear infiltrates were NTPDase1+ at Ep. Given the main role of NTPDase1 to degrade proinflammatory ATP, we hypothesized that the observed up-regulation of NTPDase1 may be associated with the transition between proinflammatory M1-like to neuroprotective M2-like phenotype of microglia/macrophages during EAE. Functional phenotype of reactive microglia/macrophages that overexpress NTPDase1 was assessed by multi-image colocalization analysis using iNOS and Arg1 as selective markers for M1 and M2 reactive states, respectively. At the peak of EAE NTPDase1 immunoreactivity showed much higher co-occurrence with Arg1 immunoreactivity in microglia and macrophages, compared to iNOS, implying its stronger association with M2-like reactive phenotype. Additionally, in ∼80% of CD68 positive cells NTPDase1 was coexpressed with Arg1 compared to negligible fraction coexpresing iNOS and ∼15% coexpresing both markers, additionally indicating prevalent association of NTPDase1 with M2-like microglial/macrophages phenotype at Ep. Together, our data suggest an association between NTPDase1 up-regulation by reactive microglia and infiltrated macrophages and their transition toward antiinflammatory phenotype in EAE.

## Introduction

Multiple sclerosis (MS) is a chronic autoimmune disease of the central nervous system (CNS), characterized by focal neurodegenerative and demyelinating lesions (Lassmann, 2018). The disease-associated tissue injury is the consequence of neuroinflammation, conducted by CNS resident reactive microglia and astrocytes, and infiltrated cells, mainly Th1, Th17 and monocytes/macrophages (Lassmann, 2014). Although MS is a uniquely human disease and despite different levels of similarities with various models of experimental autoimmune encephalomyelitis (EAE) (Behan and Chaudhuri, 2014) EAE is still the only model reflecting range of pathological and clinical features of MS (Van Der Star et al., 2012; Baker and Amor, 2014). The accumulated data obtained in EAE model, point to microglia/macrophages and astrocytes as critical players in neuroinflammation associated with MS/EAE (Mensah-Brown et al., 2011; Bjelobaba et al., 2018).

Although the causative factors of the autoimmune response in MS are not entirely known, it is considered that the pathological trigger in EAE are self-reactive CD4+ Th1 and Th17 cells, which enter the CNS, where they become reactivated by resident phagocytes (Miljkovic et al., 2011), initiating inflammatory reaction and inducing microglial and astrocytic activation. Reactive microglia and astrocytes propel neuroinflammation that results in demyelination, cell loss and axonal damage (Murphy et al., 2010; Brambilla et al., 2014). In MS, the subsequent cross-talk between reactive microglia/macrophages and activated astrocytes usually leads to self-sustained chronic neuroinflammation and progressive/irreversible neurodegeneration (Brosnan and Raine, 2013; Lassmann, 2018), whereas in the acute EAE models, the full-blown neuroinflammation turns into resolution, with ensuing myelin repair and tissue recovery. The accumulated data show that the course of the pathogenic event, chronic vs. acute, depends above all, on the functional state of activated microglia and astrocytes (Brosnan and Raine, 2013; Cherry et al., 2014).

As resident macrophages in the CNS, ramified microglia are constantly engaged in surveillance of the microenvironment, searching for signs of tissue or metabolic damage (Hanisch and Kettenmann, 2007). When they encounter harmful stimuli, microglia rapidly respond by upregulating MHC class II and co-stimulatory molecules (Becher et al., 2006), and by enhancing the production of cytokines, chemokines, reactive oxygen and nitrogen species, accompanied with shortening and thickening of processes and adoption of amoeboid morphology (Saijo and Glass, 2011). Specific cytokine milieu may polarize microglia and macrophages toward one end of the spectrum of continuous phenotypes, termed M1 and M2 (Mosser and Edwards, 2008; Cherry et al., 2014) (but see also Ransohoff, 2016). The two extreme phenotypes were characterized *in vitro*, with M1 proinflammatory phenotype induced by “classical” activation using proinflammatory factors such as IFNγ and LPS and “alternatively” activated M2 antiinflammatory phenotype, obtained after IL-4 or IL-13 exposure (Saijo and Glass, 2011). *In vivo*, activated microglia and macrophages usually acquire intermediate, overlapping phenotypes between these two extreme activation states, with functions depending on combination of M1/M2 polarization markers (Fumagalli et al., 2015; Lan et al., 2017). Although M1 and M2 phenotypes were observed only *in vitro*, M1/M2 classification is useful in understanding functions of microglia/macrophages in various CNS pathologies (Hu et al., 2015). Namely, in mouse model of spinal cord injury, despite global rise in both type of markers, majority of microglia and macrophages developed M1 phenotype (Kroner et al., 2014). In model of stroke, transition from M2 microglia/macrophages to M1 phenotype was reported (Hu et al., 2012), whereas in MS and in an animal model of epilepsy, mixed M1/M2 phenotypes were detected (Vogel et al., 2013; Benson et al., 2015; Fumagalli et al., 2015). The pathogenic context and/or dominant activated microglial phenotype further drive activation of astrocytes. Activated astrocytes may polarize toward one of two functional states, termed A1 and A2 (Liddelow et al., 2017). A1 astrocytes up-regulate gene expression of complement component 3 (C3) and proinflammatory cytokines, that determine their neurotoxic phenotype, while A2 astrocytes up-regulate gene expression of many neurotrophic factors and antiinflammatory cytokines, that determine their neuroprotective phenotype (Zamanian et al., 2012). Therefore, prevailing functional phenotypes of activated microglia and astrocytes determine the nature and course of neuroinflammation (Pekny et al., 2016; Tang and Le, 2016).

Neuroinflammation begins with the emergence of danger signals, which may be pathogen- or danger – associated molecular patterns. Much evidence has been collected showing that extracellular adenosine triphosphate (ATP), released in high concentrations from damaged or dying neurons acts as danger-associated molecular pattern (Di Virgilio et al., 2009). This nucleotide acts at a subset of P2 receptor subtypes expressed at resident cells (microglia and astrocytes), and infiltrating immune cells, to initiate the inflammatory response (Di Virgilio et al., 2009). The P2 receptor family comprises the P2X1-7, which are ATP-gated ion channels, and P2Y_1-14_, which belong to G-protein coupled receptors, activated by tri- and diphosphonucleotides, including ATP, UTP, ADP and UDP (Abbracchio et al., 2009). Upon release, extracellular ATP is catabolized by ectonucleotidase enzymes, that catalyze the sequential hydrolysis of ATP to adenosine. Ectonucleoside triphosphate diphosphohydrolases-1 and -2 (NTPDase1 and NTPDase2) and ecto-5′-nucleotidase (e-5NT/CD73), which are most abundantly expressed by the glial cells (Braun et al., 2000, 2003; Brisevac et al., 2015) are capable to complete degradation of extracellular ATP to adenosine (Yegutkin, 2008). However, each step of the nucleotide hydrolysis generates a ligand for a distinct purinergic receptor subtype (Yegutkin, 2014). NTPDase1, also known as CD39, hydrolyzes ATP and ADP equally well, thus efficiently clearing the P2 receptor ligands from the extracellular space (Kukulski and Komoszynski, 2003). In rodent brain, NTPDase1 is constitutively expressed by microglia, endothelial and smooth muscle cells of the vasculature (Braun et al., 2000), thus being involved in microglial activation (Farber and Kettenmann, 2006), regulation of blood-brain barrier function (Bynoe et al., 2015), and control of cerebral blood flow (Sevigny et al., 2002). On the other hand, NTPDase2, mostly associated with white matter astrocytes and neural progenitors (Braun et al., 2003; Jakovljevic et al., 2017), produces a ligand for ADP-sensitive P2Y_1_, P2Y_12_, and P2Y_13_ receptors present at astrocytes, microglial cells, oligodendrocytes and neurons (Abbracchio et al., 2009; Pérez-Sen et al., 2015). Finally, e-5NT/CD73, abundantly expressed by astrocytes, vascular endothelium, and choroid plexus epithelium (Jacobson and Gao, 2006; Mills et al., 2008; Lavrnja et al., 2015), generates adenosine (Zimmermann, 1992) which activates G-protein coupled adenosine receptor subtypes, A_1_, A_2A_, A_2B_, and A_3_ (Fredholm et al., 2011). It is of special note, that in contrast to ATP, which induces an inflammatory response, adenosine acts as a potent antiinflammatory and immunosuppressive factor (Di Virgilio and Vuerich, 2015). Microglia and astrocytes are well-equipped with all subtypes of purinergic receptors, and they have a central role in their dynamic modal shifts during neuroinflammation (Abbracchio and Ceruti, 2006; Koizumi et al., 2013).

A number of studies proved critical involvement of purinergic signaling in neuroinflammation associated with MS/EAE. Specifically, it has been demonstrated that e-5NT and A_2A_ receptor-mediated signaling are required for leukocyte transmigration through vascular endothelium or choroid plexus epithelium for efficient induction of EAE (Mills et al., 2012). It was also shown that NTPDase1/CD39 is essential for immunoregulatory function of specific Tregs subset of CD4+ T cells, which attenuate neuroinflammation during EAE (Wang et al., 2014). Expression of NTPDase1 in dendritic cells supress EAE development through decreasing Th1 and Th17 activation, while vaccination with IL-27 treated dendritic cells reduce severity of chronic EAE due to their induction of NTPDase1 (Mascanfroni et al., 2013). Dynamic alterations in the activity of the whole ectonucleotidase enzyme chain have been reported in EAE (Lavrnja et al., 2009), due to significant induction of e-5NT and down-regulation of NTPDase2 at mRNA and protein level, mostly by reactive astrocytes in the spinal cord of affected animals (Lavrnja et al., 2015; Jakovljevic et al., 2017). Significant induction of NTPDase1 protein has been demonstrated in EAE (Jakovljevic et al., 2017) and traumatic brain injury model, where it was upregulated by reactive microglia (Nedeljkovic et al., 2006). On the other hand, induction of NTPDase1 and e-5NT was shown *in vitro* in IL-4 stimulated macrophages exhibiting M2 phenotype (Zanin et al., 2012). Given the main function of NTPDase1 is to hydrolyze ATP and ADP, thereby providing the substrate for e-5NT and generation of adenosine, it is likely that the induction of NTPDase1 by reactive microglia/macrophages may be a part of the program that guides the development of M2-like microglial/macrophages phenotype and consequent induction of neuroprotective astrocyte phenotype (Shinozaki et al., 2017). Therefore, in the present study, we have analyzed the induction of NTPDase1 during EAE and identified microglia/macrophages as major cell types responsible for the induction. Since microglia and macrophages may polarize toward M1 or M2 reactive phenotypes, we have further established a potential association between NTPDase1 induction and polarization of microglia/macrophages to neuroprotective M2-like phenotype.

## Materials and Methods

### Experimental Autoimmune Encephalomyelitis and Disease Score Assessment

Eight-week old female rats of Dark Agouti (DA) inbred strain (48 animals) from the local colony were used for the experiments. Experimental protocols were approved by the Ethical Committee for the Use of Laboratory Animals of the Institute of Biological Research “Siniša Stanković,” Belgrade, Serbia (Application No.01-11/14) and in compliance with the ECC Directive (2010/63/EU) on the protection of animals used for experimental and other scientific purposes. Littermate animals were housed 2–5 per cage under conventional conditions: constant temperature and humidity, 12 h light/dark cycle, and laboratory chow and water *ad libitum*. Paralyzed animals were fed and given water manually. Animals were immunized on the same day (36 animals). Immunization was performed under anesthesia in the hind footpad by a subcutaneous injection of 150 μl of encephalitogenic emulsion of rat spinal cord homogenate (50% *w/v* in saline) mixed with an equal volume of complete Freund’s adjuvant containing 1 mg/mL *Mycobacterium tuberculosis* (CFA; Sigma, St. Louis, MO, United States). Age-matched animals (12 animals) were anesthetized without subsequent immunization and used as an intact physiological control. All the animals were weighed and monitored daily for the clinical signs of EAE up to 30 days post immunization. Disease severity was assessed by standard 0–5 EAE grading scale as follows: 0 – unaffected; 0.5 – reduced tail tone; 1 – tail atony; 1.5 – slightly/moderately clumsy gait, impaired righting ability or combination; 2 – hind limb paresis; 2.5 – partial hind limb paralysis; 3 – complete hind limb paralysis; 3.5 – complete hind limb paralysis with fore limb weakness; 4 – moribund state and 5 –death of the animal. Scores obtained by blind scoring were averaged and plotted as daily mean clinical score.

### Tissue Preparation

The animals were euthanized at three time points representing three phases of the acute monophasic disease – onset (Eo), peak (Ep), and end of symptoms (Ee). Under deep anesthesia with Zoletil^®^ 50 (Virbac, France; 30 mg/kg i.p.), animals were perfused with 0.9% sodium chloride and decapitated. Following decapitation, lumbosacral spinal cords were isolated and prepared for RNA isolation, Western blot, Flow Cytometry or cryosectioning.

### Real-Time PCR

Lumbosacral part of spinal cord (3/group) was cut and used for RNA isolation with TRIzol^®^ reagent (Invitrogen, Carlsbad, CA, United States) according to manufacturer’s instructions. RNA concentrations were measured using spectrophotometer and RNA purity was determined by measurement of A_260_/A_280_ and A_260_/A_230_ ratios. A volume equivalent to 1 μg of RNA was used for reverse transcription with High Capacity cDNA Reverse transcription kit (Applied Biosystems, Foster City, CA, United States). cDNA was then diluted 10 times and these probes were used for real-time PCR standard protocol described previously (Jakovljevic et al., 2017) with QuantStudioTM 3 Real-Time PCR System (Applied Biosystems, Foster City, CA, United States). For negative control, cDNA template was omitted from PCR mixture. Relative expression of target gene was determined by 2^-ΔΔCt^ method, with *Gapdh* (glyceraldehide-3-phosphate dehydrogenase) as reference gene (Lavrnja et al., 2015). Quality control was routinely performed by melting curve analysis and gel electrophoresis of obtained PCR products. Primer sequences are listed in Table 1.

**Table 1:** Primer sequences.

Target gene	Forward	Reverse
*Gapdh*	TGGACCTCATGGCCTACAT	GGATGGAATTGTGAGGGAGA
*Entpd1*	TCAAGGACCCGTGCTTTTAC	TCTGGTGGCACTGTTCGTAG
*Aif1*	CCAGCGTCTGAGGAGCTATG	CGTCTTGAAGGCCTCCAGTT
*Itgam*	GACTCCGCATTTGCCCTACT	TGCCCACAATGAGTGGTACAG
*Cx3cr1*	TTCTTCCTCTTCTGGACGCCT	TGAGGCAGCAGTGGCTAAAC
*Nos2*	CAGCCCTCAGAGTACAACGAT	CAGCAGGCACACGCAATGAT
*Arg1*	TAACCTTGGCTTGCTTCGG	GTGGCGCATTCACAGTCAC
*Cd68*	TGTGTGTCTGACCTTGCTGG	AAGGATGGCAGAAGAGTGGC


### Isolation of Crude Plasmamembrane Fraction

Lumbosacral spinal cord parts for each group (3/group) were pooled and further processed to obtain crude plasmamembrane preparation (Gray and Whittaker, 1962). After homogenization in 5 mmol/L Tris, pH 7.4 buffer containing 320 mmol/L sucrose, the homogenates were centrifuged at 3.000 × *g* for 10 min at 4°C. Supernatant was taken and further centrifuged at 12.000 × *g* for 40 min at 4°C. Then, the obtained pellets were resuspended in 5 mmol/L Tris pH 7.4 and used for Western blot, after determination of protein concentration using Micro BCA Protein Assay Kit (Thermo Fisher Scientific, Rockford, IL, United States).

### Western Blot

Crude plasma membrane samples were diluted in 4 × Laemmli Sample Buffer (#161-0747 Bio-Rad, Hercules, CA, United States) and incubated for 5 min at 95°C. After cooling on ice, sample proteins (15 μg per lane) were loaded on a 7.5% PAGE-SDS gels in non-reducing conditions and the resolved proteins were electrotransferred to a support membrane (Immobilon-P transfer membrane, Millipore, Darmstadt, Germany). After transfer, membranes were blocked in 5% Bovine Serum Albumine (BSA, Sigma, St. Louis, MO, United States) in Tris-buffer saline Tween-20 (TBST) and probed with anti-NTPDase1 primary antibodies indicated in Table 2, at 4°C overnight. After triple washing in TBST for 10 min, the membranes were incubated in anti-guinea pig secondary HRP-conjugated IgG antibodies indicated in Table 2, 2 h/room temperature. X-ray films (Kodak, Rochester, NY, United States) were used for visualization of chemiluminescent signal (ECL, GE Healthcare, United States). Densitometric analysis using *ImageJ* software package (**RRID:SCR_003070**) was performed to determine optical density of protein bands. Optical density of β-actin bands in the same lane was used for normalization and resulting values obtained from 5 separate membranes were expressed relative to control (100%) ± SEM.

**Table 2:** List of antibodies.

Antibody	Source and type	Dilution	Manufacturer
NTPDase1 mN1-2C(I4,I5)	Guineapig, polyclonal	1:200 (IF)^a^	Ectonucleotidases-ab.com;Cat# NTPDase1
		1:6000 (WB)^b^	
		1:400 (FACS)^c^	
GFAP	Mouse, monoclonal	1:500 (IF)	UC Davis/NIH NeuroMab Facility (73–240), RRID:AB_10672298ED1
		1:200 (IHC)^d^	
Iba1	Goat, polyclonal	1:400	Abcam ab5076, RRID:AB_2224402
CD68/ ED1	Mouse, monoclonal	1:100	Abcam ab31630, RRID:AB_1141557
iNOS	Rabbit, polyclonal	1:200	Abcam ab15323, RRID:AB_301857
Arg-1	Rabbit, polyclonal	1:200	Sigma AV45673, RRID:AB_1844986
Glutamine synthetase	Rabbit, polyclonal	1:1000	Abcam ab49873, RRID:AB_880241
Vimentin	Mouse, monoclonal	1:200	Dako M0725 RRID:AB_10013485
COX-2	Goat, polyclonal	1:50	Santa Cruz sc-1745, RRID:AB_631309
C3	Goat, polyclonal	1:500	Thermo Fisher Scientific PA1-29715 RRID:AB_AB_2066730
NF-H/SMI32	Mouse, monoclonal	1:2000	Covance Research Products Inc AB_509997, RRID:AB_509997
CD4	Mouse, monoclonal	1:100	Sigma-Aldrich SAB4700733, RRID:AB_476825733
CD40	Rabbit, polyclonal	1:100	Abcam ab65853 RRID:AB_1141079
CD45	Mouse, monoclonal Conjugated to PE	1:10	Bio-Rad MCA43PE RRID:AB_321412
P2Y_1_	Rabbit, polyclonal	1:200	Alomone Labs; #APR-0009, RRID:AB_2040070
Anti-mouse IgG Alexa Fluor 488	Donkey, polyclonal	1:250	Invitrogen A21202 RRID:AB_141607
Anti – guinea pig IgG Alexa Fluor 555	Goat, polyclonal	1:250	Invitrogen A-21435 RRID:AB_2535856
Anti – guinea pig IgG Alexa Fluor 488	Goat, polyclonal	1:200 (FACS)	Invitrogen A-11073 RRID: AB_2534117
Anti-goat IgG Alexa Fluor 488	Donkey, polyclonal	1:250	Invitrogen A-11055 RRID:AB_142672
Anti-rabbit IgG Alexa Fluor 488	Donkey, polyclonal	1:250	Invitrogen A-21206, RRID:AB_141708
Anti-mouse IgG Alexa Fluor 350	Donkey, polyclonal	1:250	Invitrogen A10035 RRID:AB_2534011
Anti-goat IgG Alexa Fluor 350	Donkey, polyclonal	1:250	Invitrogen A-21081 RRID:AB_2535738
Anti-mouse HRP conjugated IgG	Donkey, polyclonal	1:250	Santa Cruz sc-2314, RRID:AB_641170
Anti-rabbit HRP conjugated IgG	Donkey, polyclonal	1:250	Santa Cruz sc-2313 RRID:AB_641181


**^*a*^IF, immunofluorescence; ^*b*^WB, western blot; ^*c*^FACS, flow cytometry; ^*d*^IHC, immunohistochemistry.**

### Flow Cytometry

Lumbosacral parts of spinal cords (3/group) were homogenized through nylon sieve and centrifuged at 800 × *g* for 3 min. Pellet was resuspended in 30% Percoll (Sigma-Aldrich, United States), transferred gently to a tube filled with 70% Percoll and centrifuged at 870 × g for 50 min at room temperature. Mononuclear infiltrate cell layer was transferred and resuspended in PBS and centrifuged again at 900 × g for 10 min. Pellet was re-suspended in PBS, cells were counted and 10^6^ cells were used for subsequent labeling and flow cytometric analysis. Cells were again centrifuged at 3000 × *g* for 4 min, re-suspended in PBS with 10% normal rat serum and incubated with primary antibodies (Table 2) for 45 min at 4°C. Cells were washed three-times in PBS and then incubated with fluorescently labeled secondary antibody, where primary antibody was unlabeled (Table 2). After triple washing step in PBS, flow cytometric analysis was performed on CyFlow^®^ Space Partec (Partec GmbH, Munster, Germany), and the software used for data analysis was PartecFloMax^®^ (Partec GmbH, Munster, Germany).

### Immunohistochemistry and Immunofluorescence

Transverse cryosections of the lumbar spinal cord (20 μm thickness) (3 animals/group) were incubated at room temperature for 30 min for acclimatization prior to triple washing step in PBS in duration of 5 min. For immunohistochemistry, sections were subsequently incubated in 0.3% hydrogen peroxide in methanol for blocking the endogenous peroxidase. Sections were then incubated for 1 h in blocking solution containing either 1% BSA or Normal Donkey Serum (10% in PBS; Santa Cruz Biotechnology, Santa Cruz, CA, United States), followed by overnight incubation with primary antibodies to various CNS cell type specific markers (Table 2) at 4°C in a humid chamber. After the first primary antibody incubation and subsequent triple PBS washing step, the sections were incubated with secondary antibody linked with horseradish peroxidase (HRP) for immunohistochemistry or fluorescent label for immunofluorescence, respectively (Table 2), for 2 h at room temperature in a humid chamber in the dark and washed three times in PBS. In immunohistochemistry method, specific antigen – antibody reaction was visualized by brief incubation of sections with 3,3′S-diaminobenzidine-tetrahydrochloride (DAB, Dako, Glostrup, Denmark). Reaction was stopped by washing in tap water, followed by 5 min incubation of sections in 70, 96, 100% ethanol and twice in xylene, respectively for dehydration. Sections were mounted with DPX Mounting medium (Fluka, Buchs, Switzerland) and examined under a Zeiss Axiovert microscope (Carl Zeiss GmbH, Vienna, Austria). For double and triple immunofluorescence, blocking and overnight primary antibody incubation steps were repeated. Nuclei were counterstained with Hoechst 33342 dye (5 μg/ml – Life Technologies, Invitrogen, Carlsbad, CA, United States) for 10 min at room temperature and washed in PBS to minimize the background. Sections incubated without primary antibody were used as negative controls. Microscopic slides with cryosections for immunofluorescence were mounted in Mowiol (Calbiochem, Millipore, Germany) and captured on Zeiss Axiovert fluorescent microscope (Zeiss, Jena, Germany).

### Quantification of Immunofluorescence and Multi-Image Colocalization Analysis

Image analyses were conducted using ImageJ open-source platform^1^ (**RRID:SCR_003070**). The number of infiltrates per spinal cord cross section and the number of cells per infiltrate were counted manually from *n* ≥ 6 sections per spinal cord, from 3 animals per disease phase, from two separate EAE experiments. Raw immunofluorescent micrographs taken under the same conditions were used to measure integrated fluorescence density and the density confined within five pre-defined regions of interest (ROIs), with background fluorescence subtraction for at least 3 images per ROI and *n* ≥ 6 sections per spinal cord, from 3 animals per experimental time point. The ROI were dorsal gray matter, ventral gray matter, dorsal white matter, ventral white matter, and lateral white matter. Fluorescence intensity corresponding to NTPDase1 of α-motoneuronal somata was determined as density confined within ROI, determined as an outline of motor neuron bodies, from at least 3 ROI per section, for *n* ≥ 6 sections per spinal cord, from 3 animals per experimental time point. Multi-image colocalization analysis was performed in the whole section, both in the white and gray matter, using JACoP ImageJ plugin. A degree of overlap and correlation between multiple channels was estimated by calculating Pearson’s correlation coefficient (PCC) and Manders’ colocalization coefficient (MCC) (Dunn et al., 2011). PCC is statistical parameter that reflects both co-occurrence (degree at which intensities of two channels for each pixel are beyond or above the threshold), and correlation (pixel-for-pixel proportionality in the signal levels of the two channels). MCC is sensitive just to co-occurrence and it measures the fraction of pixels with positive values for both channels, regardless of the signal intensities. When determining the fractional overlap between two signals, signal 1 and signal 2, two different MCC values were derived. *MCC*_1_ reflects the fraction of signal 1 that co-localizes with signal 2, while *MCC*_2_ represents the fraction of signal 2 that co-localizes with signal 1.

### *In situ* Hybridization

Cryosections of the lumbosacral spinal cord were incubated with 15 μg/ml Proteinase K solution (Invitrogen, Carlsbad, CA, United States) in a buffer containing 5 mmol/L Tris-HCl, 1 mmol/L EDTA and 1 mmol/L NaCl in diethyl-pyrocarbonate (DEPC; Sigma, St. Louis, MO, United States) treated water at 37°C for 20 min in a humid chamber prior to PBS washing step and brief dehydration in a series of ethanol solutions with ascending concentrations. After dying, sections were covered with *in situ* hybridization buffer (50% formamide, 20% 20x Saline Sodium Citrate Buffer – SSC, 5% Phosphate Buffer and 10 μg/ml Deoxyribonucleic acid from herring sperm – Sigma, St. Louis, United States) containing 1000 pg/μl of digoxigenin (DIG) – labeled NTPDase1 oligonucleotide probe and incubated overnight in humid chamber at 42°C. The sequence of NTPDase1 oligonucleotide probe was GCTGGATGCCGGGTCGTCTCACA CCAACCTGTACATCTAC. The probe was labeled with DIG Oligonucleotide 3′ – End Labeling Kit, 2nd Generation (# 03 353 575 910, Roche, Basel, Switzerland) according to manufacturer’s instructions. Negative controls contained no probe in hybridization buffer. On the following day the sections were washed in 5×, 1×, and 0.2× SSC buffer (3 mol/L NaCl, 0.3 mol/L Na-citrate, pH 7), twice at 42°C and once in PBS at room temperature. Blocking step consisted of incubation in blocking buffer with final concentration of 0.01% Fetal Calf Serum (FCS, Invitrogen, Carlsbad, CA, United States) and BSA at room temperature for 20 min. The sections were then incubated with Anti–DIG Alkaline Phosphatase conjugated antibody (1:200, #011093274910, Roche, Basel, Switzerland) for 2 h at room temperature and washed in 0.1% Tween-20 in PBS. Visualization step was done with NBT/BCIP (#011697471001, Roche, Basel, Switzerland) according to manufacturer’s instruction with 0.2 μmol/L levamisole (Sigma, St. Louis, MO, United States). Slides with spinal cord sections were then used for HRP – based immunohistohemical counterstaining of GFAP. All reagents for *in situ* hybridization were made in DEPC-treated water and sterilized.

### Data Analysis

Data homogeneity was tested using Levene’s test (SPSS 20, IBM, Armonk, NY, United States). Statistical analysis was performed in GraphPad Prism 5 Software^®^ (GraphPad Software, La Jolla, CA, United States) using Kruskal – Wallis test followed by Dunn’s *post hoc* test or one-way analysis of variance (ANOVA) followed by Dunnett *post hoc* test, as apropriate. The data were presented as Mean ± SEM and considered statistically significant for *p* < 0.05.

## Results

### Disease Score During EAE

**FIGURE 1:**
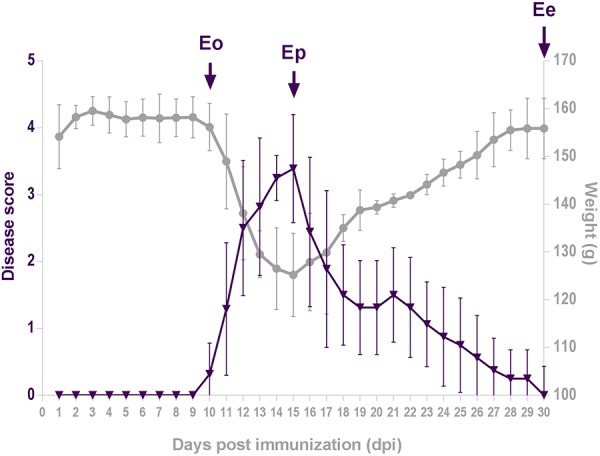
Disease score. Animals were weighed and scored daily for neurological signs of EAE using standard 0–5 EAE grading scale. Plots represent daily mean disease score ± SEM (violet triangles) and mean body mass (gray circles) during EAE, measured in two separate rounds of EAE inductions. Arrows point to days 10, 15, and 30 post-immunization, which correspond to the onset of disease (Eo), the peak of neurological symptoms (Ep) and the end of disease (Ee).

The disease initiated by immunization with the encephalitogenic emulsion exhibited acute monophasic course in all animals (Figure 1). The severity of the disease at each day post-induction (dpi) was expressed as mean disease score that was calculated using arbitrary 1–5 disease scale. The daily mean disease score which reflected the severity of symptoms, began to increase from 10 dpi (Eo), peaked at 15 dpi (Ep) and thereafter gradually decreased and ended at 30 dpi (Ee). Changes in animal body weight mirrored the disease score, showing maximum weight loss at the peak of the disease.

### Expression of NTPDase1 During EAE

**FIGURE 2:**
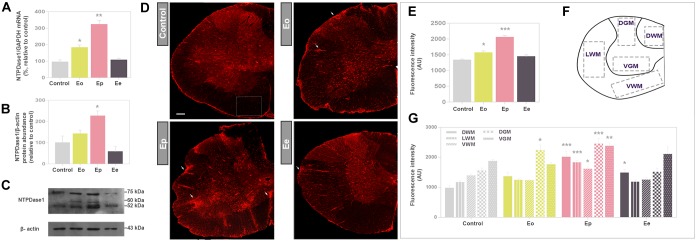
Expression analysis of NTPDase1 during EAE. **(A)** Transcriptional expression of *Entpd1* in spinal cord tissue homogenate. Bars represent mean NTPDase1/GAPDH-mRNA abundance (±SEM), determined in total RNA isolated from the lumbar spinal cord of control rats (100%) and rats at Eo, Ep, and Ee. Samples were collected from three animals per group, from two separate experiments. ^∗^*p* < 0.05, ^∗∗^*p* < 0.001. **(B)** Western blot analysis of NTPDase1 in crude plasma membranes isolated from spinal cord tissue. The intensities of protein bands were assessed by densitometric measurements using ImageJ software and expressed relative to the optical density (OD) of the β-actin band in the same lane (NTPDase1 protein/β-actin ratio). The NTPDase1/β-actin value obtained for the control sample was defined as 100% ± SEM and the ratios obtained for other samples were expressed relative to the control (bars). Bars represent the mean NTPDase1 protein abundance (±SEM) from *n* ≥ 4 determinations performed on two independent sample preparations. Significance inside the graph: ^∗^*p* < 0.05. **(C)** Representative support membrane showing the position of bands corresponding to NTPDase1/CD39, visualized on X-Ray films with the use of chemiluminescence. **(D)** NTPDase1 immunofluorescence, showing the pattern of immunoreaction at control sections, and sections obtained during EAE. Arrows indicate the position of infiltrates. Scale bar applicable to all micrographs = 200 μm. **(E)** Integrated fluorescence densities expressed in arbitrary units (AU), corresponding to NTPDase1, obtained from control sections and during EAE. Bars represent mean AU ± SEM from *n* ≥ 6 sections per animal, from 3 animals per experimental group, from two separate experiments. ^∗^*p* < 0.05, ^∗∗∗^*p* < 0.0001 in comparison to control. **(F)** Regions of interest (ROIs) used for quantitative analysis of NTPDase1-immunofluorescence intensity (DGM, dorsal gray matter; VGM, ventral gray matter; DWM, dorsal white matter; VWM, ventral white matter; LWM, lateral white matter). **(G)** Mean integrated fluorescence density of NTPDase1-immunofluorescence (AU) corresponding to ROIs. Bars represent mean ± SEM from *n* ≥ 4 micrographs per ROI per section, from *n* ≥ 6 sections per animal, from 3 animal per group, from two separate experiments. Significance: ^∗^*p* < 0.05, ^∗∗^*p* < 0.001 and ^∗∗∗^*p* < 0.0001 compared to control. **(A,E,G)** Kruskal–Wallis with Dunn’s *post hoc* test, **(B)** one-way ANOVA with Dunnet’s *post hoc* test.

The expression profile of NTPDase1 at mRNA (*Entpd1)* and protein level in the lumbar spinal cord in control and EAE animals are presented in Figure 2A–C. Both mRNA and protein expression gradually increased during EAE, with most pronounced induction at Ep. At mRNA level, NTPDase1 expression increased two-fold at Eo and three-fold at Ep while protein expression increased two-fold at Ep. At the end of the disease, both mRNA and protein abundance returned to the control levels.

Expression of NTPDase1 in spinal cord cross sections was visualized by fluorescence immunohistochemistry (Figure 2D), using well-characterized antibodies directed to NTPDase1 (mN1-2C) (Martin-Satue et al., 2009). In control sections under low magnification, the most intensive NTPDase1 immunoreactivity was associated with the pial surface, numerous small ramified cells in the gray matter and elongated fibrous elements which traverse the white matter. The overall fluorescence intensity slightly increased at ***Eo*** and markedly increased at ***Ep***, particularly at NTPDase1 positive cellular infiltrates and fibrous elements at ***Ee***. The intensity of the fluorescence signal demonstrated a significant increase in total NTPDase1 immunoreactivity at ***Eo*** and ***Ep*** relative to control (Figure 2E), while determination of pixel intensities confined within individual regions (Figure 2F), revealed a significant increase in NTPDase1 immunoreactivity in dorsal gray matter at ***Eo***, in all regions at ***Ep***, and in dorsal white matter at ***Ee*** (Figure 2G).

### Visualizing Cells That Express Entpd1 mRNA During EAE

**FIGURE 3:**
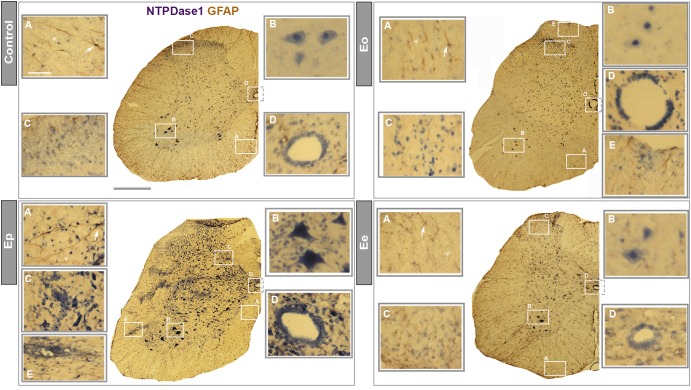
*In situ* NTPDase1-mRNA hybridization. *In situ* hybridization using NTPDase1-specific DIG-labeled oligonucleotide probes in a combination with HRP - based immunohistohemical staining and GFAP immunohistochemistry. Low-power magnification micrographs show distribution of NTPDase1-mRNA at cross-sections from control (scale bar applicable to all low-power micrographs = 500 μm), Eo, Ep, and Ee. High-power micrographs are magnified from the areas enclosed by rectangles at each low-power micrograph. Scale bar at **(A)** insertion from control section applicable to all insertions = 50 μm. NTPDase1 mRNA expression is visible in microglia-like cells (indicated by arrowheads) and astrocytes (indicated by arrows) in the white matter **(A)**, large α-motoneurons in ventral horns of gray matter **(B)**, neurons in dorsal gray matter **(C)**, ependymal cells **(D)**, perivascular infiltrates and vascular endothelial cells **(E)**.

Identification of the cell types expressing NTPDase1 during EAE was assessed by *in situ* hybridization of *Entpd1* mRNA, combined with immunohistochemical counterstaining directed to GFAP (Figure 3). In control sections under higher magnification, *Entpd1* mRNA was detected in GFAP immunoreactive astrocytes in the white matter, in small ramified cells distributed throughout the cross-section, in large motoneurons in the ventral gray matter, in vascular endothelial cells and in ependymal cells lining the central canal. At ***Eo*** increased staining was observed at ependymal cells and small number of infiltrated cells. The staining pattern markedly changed at ***Ep***, when prominent *Entpd1* mRNA labeled numerous ovoid cells resembling activated microglia, scattered in all areas of the spinal cord. The staining was also more pronounced in numerous infiltrated mononuclear cells, motoneurons in ventral gray matter, vascular endothelial and ependymal cells. At ***Ee***, the overall pattern of *Entpd1* mRNA *in situ* hybridization was comparable with the control. At all time-points association of *Entpd1* mRNA with GFAP immunoreactive astrocytes in the gray matter was weak and indistinct.

### Expression of NTPDase1 by Microglia/Macrophages and Astrocytes

**FIGURE 4:**
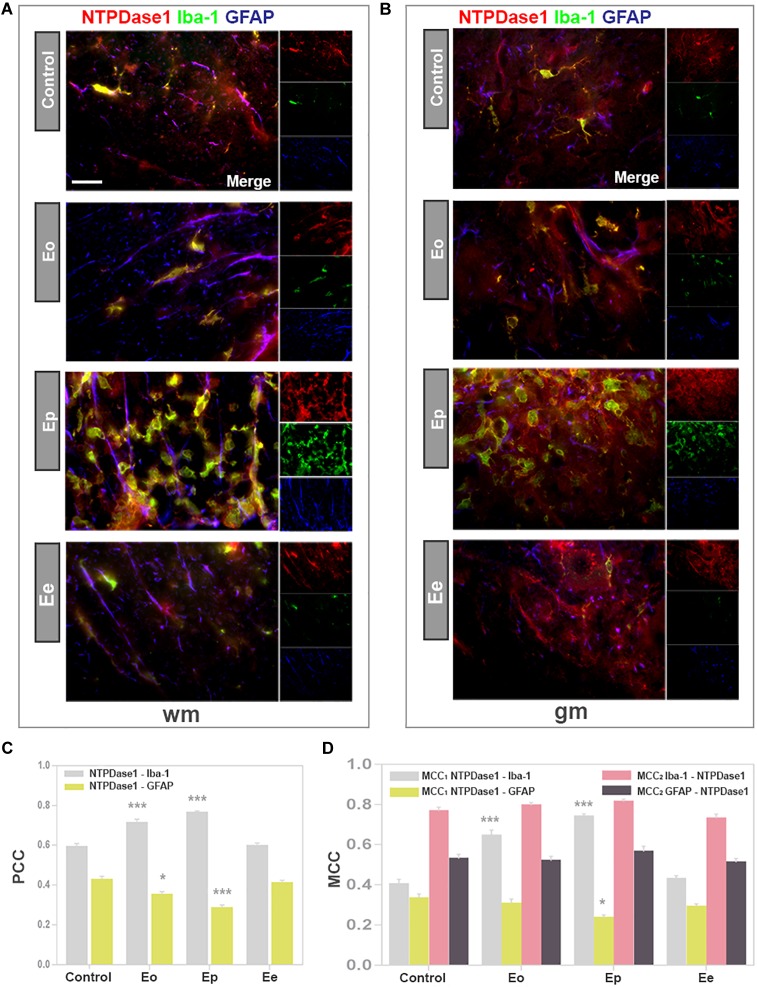
Expression of NTPDase1 by microglia/macrophages and astrocytes. **(A,B)** Representative micrographs of spinal cord cross-sections from white matter (wm) and gray matter (gm), respectively, showing triple immunofluorescence labeling directed to NTPDase1 (*red fluorescence*), Iba1 (*green fluorescence*) and GFAP (*blue fluorescence*). Scale bar applicable to all micrographs = 20 μm. **(C)** Pearson’s correlation coefficients (PCC), showing level of overlap between NTPDase1/Iba1-*ir* (gray bars) and NTPDase1/GFAP-*ir* (yellow bars) in the white and gray matter of spinal cord cross-sections. Bars represent mean PCC ± SEM from *n* ≥ 4 micrographs per section, for *n* ≥ 6 sections per animal, from 3 animals per experimental group, from two separate experiments. Significance inside the graph:^∗^*p* < 0.01, ^∗∗∗^*p* < 0.0001 in comparison to control. **(D)** Manders’ colocalization coefficients *MCC*_1_ and *MCC*_2_, showing fractional overlap between pairs of signals as shown inside the graph, obtained for the whole spinal cord cross-section. Bars represent mean ± SEM from *n* ≥ 4 micrographs per section, *n* ≥ 6 sections per animal, from 3 animals per experimental group, from two separate experiments. Significance inside the graph: ^∗^*p* < 0.01, ^∗∗∗^*p* < 0.0001 in comparison to control. **(C,D)** Kruskal–Wallis with Dunn’s *post hoc* test.

Contributions of microglia/macrophages and astrocytes to the induction of NTPDase1 in lumbar spinal cord during EAE were evaluated by triple immunofluorescence labelings directed to NTPDase1, Iba1 as constitutive marker of microglia/macrophages and GFAP as pan astrocytic marker (Figure 4). In control sections, NTPDase1 was associated with highly ramified Iba1 positive cells, that show non-overlapping pattern of tissue distribution, indicating resting microglia. At ***Eo*** the NTPDase1 immunoreactivity was located at Iba1 positive perivascular and subpial infiltrates and parenchymal cells with shortened and thickened processes and decreased intercellular distances indicating microglial activation (Figure 4A,B). ***Ep*** sections displayed significantly more double NTPDase1/Iba1 immunoreactive cells compared to control, mostly with amoeboid morphology indicative of activated microglia/macrophages and usually observed in clusters. Double reactive NTPDase1/Iba1 ovoid cells were often seen in close apposition with GFAP immunoreactive astrocytes. At ***Ee***, number of Iba1 immunoreactive cells returned to control level. Majority of Iba1 positive cells acquired ramified morphology, indicating microglial deactivation. The extent of colocalization between NTPDase1 and Iba1 immunoreactivity in the spinal cord cross-sections was assessed by determining the values of PCC and MCC. The PCC, which reflects both co-occurrence and correlation between NTPDase1 and Iba1 reactivity, increased significantly from 0.6 in control sections to almost 0.8, in ***Ep*** sections (Figure 4C), indicating significant overlap between the two fluorescence signals. The co-occurrence of two signals was expressed also in terms of contribution of NTPDase1 to the total NTPDase1/Iba1 immunoreactivity (*MCC*_1_), and contribution of Iba1- to the total NTPDase1/Iba1 immunoreactivity (*MCC*_2_). *MCC*_1_ value increased from 0.4 in control sections, to 0.8 in ***Ep*** sections, and declined to the control level at ***Ee***. The value of *MCC*_2_, which reflects the fraction of Iba1 immunofluorescence colocalized with NTPDase1, did not change during EAE (Figure 4D). Thus, the variations in *MCC*_1_ implied a significant contribution of reactive microglia and macrophages to the observed up-regulation of NTPDase1 during EAE, showing that the prominent increase in fraction of NTPDase1 immunofluorescence colocalized with Iba1 was up to 80%.

In accordance with results obtained by *in situ* hybridization, NTPDase1 immunofluorescence was observed in association with GFAP positive processes mostly in the white matter. The extent of NTPDase1/GFAP colocalization was determined by assessing both PCC and MCC. PCC values for NTPDase1/GFAP co-occurrence accounted for less than 0.4, thus confirming our microscopic observations that there is only partial association of the two signals and low basal expression of NTPDase1 at spinal cord astrocytes. The fractional overlap between NTPDase1 and GFAP immunofluorescence, expressed as *MCC*_1_ value, decreased from 0.3 in control sections to 0.2 in ***Ep*** sections (Figure 4D), implying that reactive astrocytes did not up-regulate expression of NTPDase1 during EAE. The degree of colocalization for both cell markers with NTPDase1 was additionally confirmed with preliminary results obtained by confocal microscopy (Supplementary Figure 1).

### Functional State of NTPDase1 Positive Microglia/Macrophages

While gene and protein expression analysis revealed a significant up-regulation of NTPDase1 in spinal cord tissue during EAE, immunohistochemical and colocalization data pointed to reactive microglia/macrophages as cells responsible for the progressive NTPDase1 expression during EAE. Considering that the resolution of the acute neuroinflammation is associated with the transition from pro- to antiinflammatory functional state of microglia/macrophages, it was tempting to speculate that induction of NTPDase1 by reactive microglia/macrophages may be part of the program that characterizes the M2-like phenotype. Therefore, we first assessed the expression profiles of several factors, that are well-known indicators of immune environment of the tissue during EAE (Table 3). The expression of target genes coding for Iba1 (*Aif1*), inducible nitric oxide synthase (*Nos2*), arginase1 (*Arg1*), CD68 (*Cd68*), CD11b (*Itgam*), and fractalkine receptor (*Cx3cr1*) exhibited several-fold increase, that peaked at ***Eo*** for *Nos2* and *Arg1* and at ***Ep*** for all the other analyzed genes.

**FIGURE 5:**
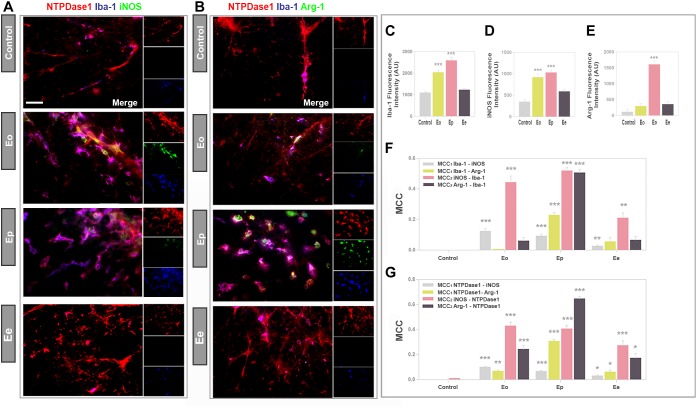
The functional state of NTPDase1-positive microglia/macrophages. **(A,B)** Representative micrographs showing triple immunofluorescence labeling directed to NTPDase1 (*red fluorescence*), Iba1 (*blue fluorescence*) and iNOS or Arg1 (*green fluorescence*) at cross-sections of lumbar spinal cord white matter in control and during EAE. Integrated density corresponding to Iba1 **(C)**, iNOS **(D)**, and Arg1 **(E)** immunofluorescence in control sections, and during EAE. Bars represent mean fluorescence ± SEM, from *n* ≥ 4 images per section, *n* ≥ 6 sections per animal, from 3 animals per experimental group, from two separate experiments. Significance inside the graph: ^∗∗∗^*p* < 0.0001 in comparison control. **(F)** MCC values obtained from multi-image colocalization analyses of micrographs presented in **(A)**, showing level of co-occurrence of pair of signals, as indicated inside the graph. ^∗∗^*p* < 0.01, ^∗∗∗^*p* < 0.0001 in comparison to control. **(G)** MCC values calculated for pairs of signals, as indicated inside the graph. Bars at mean MCC value ± SEM from *n* ≥ 4 images per section, from *n* ≥ 6 sections per animal, from 3 animals per experimental group, from two separate experiments. Significance inside the graph: ^∗^*p* < 0.05, ^∗∗^*p* < 0.001, ^∗∗∗^*p* < 0.0001 in comparison to control. **(C–G)** Kruskal–Wallis with Dunn’s *post hoc* test.

Next, we performed triple immunofluorescence labeling to assess the co-occurrence of NTPDase1/Iba1 immunoreactivity with either iNOS or Arg1, which are selective markers of M1- and M2 microglial/macrophages state respectively (Figure 5A,B). In accordance with increased mRNA expression, and observed increase in the number of Iba1 positive cells in spinal cord cross- sections, Iba1 immunofluorescence increased about 2- and 2.5-fold at Eo and Ep (Figure 5C). The overall fluorescence intensity of iNOS reactivity increased from control to ***Eo*** and ***Ep*** and then declined at ***Ee*** (Figure 5A), that is in accordance with the iNOS mRNA expression analysis. Microscopic observations were additionally confirmed by quantification of total iNOS immunofluorescence (Figure 5D). At ***Eo***, iNOS immunofluorescence considerably overlapped with Iba1- and NTPDase1 reactivity. At ***Ep***, iNOS immunoreactivity was localized both at NTPDase1-/Iba1 immunoreactive cells, and at NTPDase1 reactive/Iba1 negative ovoid cells, probably belonging to infiltrated lymphoid cells. Although the results of Arg1 expression demonstrated the strongest induction of *Arg1* mRNA at ***Eo*** (Table 2), the Arg1- immunoreactivity was observed mostly at ***Ep***, at a number of amoeboid cells that co-expressed NTPDase1 and Iba1 (Figure 5B). Accordingly, total Arg1 immunofluorescence was significantly increased only at Ep (Figure 5E). The determination of *MCC*_1_ coefficient for Iba1/iNOS co-occurrence at different phases of EAE revealed that the fraction of Iba1-immunofluorescence colocalized with iNOS was about 10% at ***Eo*** and ***Ep***, while it decreased to about 5% at ***Ee*** (Figure 5F). The *MCC*_1_ coefficient for Iba1/Arg1 immunoflurescence showed insignificant overlap between two signals at ***Eo***, then about 25% of overlap at ***Ep*** and less than 5% overlap at ***Ee*** (Figure 5F). The overlap of both reactive microglia/macrophages markers with Iba1 fluorescence declined at ***Ee***, which is in accordance with the resolution of the acute disease. Together, these data indicate that at ***Eo***, M1-like microglial/macrophages phenotype prevails, whereas, at ***Ep***, M2-like phenotype prevails. Since activated microglia/macrophages *in vivo* may simultaneously express markers of M1 and M2 activation state, we stained consecutive sections for Iba1/NTPDase1 and either iNOS or Arg1. At representative micrographs obtained by overlay of Iba1/iNOS/NTPDase1 immunofluorescence with Arg1 immunofluorescence (Supplementary Figure 2) approximately half of Iba1/NTPDase1 positive cells showed coexpression of iNOS and Arg1 at Eo, indicating mixed M1/M2 phenotype. At Ep number of iNOS/Arg1 double-positive cells decreased and only a small number of such cells per section were visible. At Eo the majority of cells showed either iNOS or Arg1 immunoreactivity, while at Ep there were only a few iNOS single positive cells and much more cells showing Arg1 immunoreactivity, indicating prevalence of M2-like microglial/macrophages activation state at the peak of EAE. However, due to overcrowding of infiltrates with microglia/macrophages, it was hard to distinguish individual Iba1 positive cells and to determine the number of Iba1 positive cells that express either iNOS, Arg1 or both markers.

The colocalization coefficients for fractional overlap between NTPDase1/iNOS and NTPDase1/Arg1 immunoreactivity confirmed that less than 10% of total NTPDase1 fluorescence overlapped with iNOS reactivity at ***Eo*** and ***Ep*** (Figure 5G), whereas 10 and 30% of NTPDase1fluorescence overlapped with Arg1 at ***Eo*** and ***Ep***, respectively. These data confirm significantly higher overlap of NTPDase1- and Arg1 immunoreactivity at ***Ep***, that suggest an association of NTPDase1 with M2-like microglia/macrophages. The MCC for Arg1/NTPDase1 co-occurrence implies a significant overlap between Arg1 and NTPDase1 immunofluorescence of about 65% at ***Ep***.

### Expression of NTPDase1 by Mononuclear Infiltrates

**FIGURE 6:**
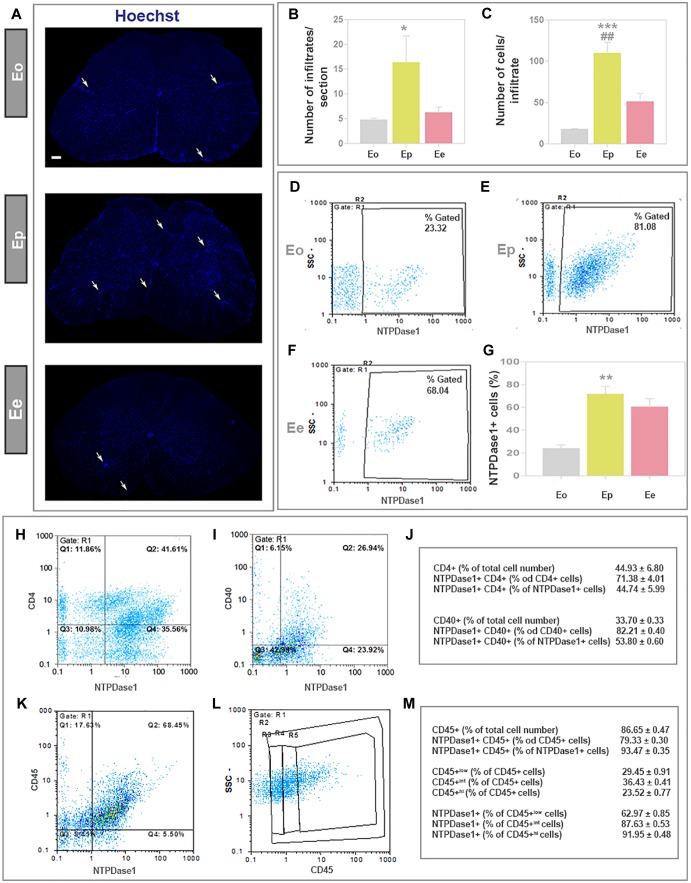
Expression of NTPDase1 by mononuclear cell infiltrates. **(A)** Low-power micrographs of spinal cord cross-sections stained by Hoechst for cell nuclei. Arrows indicate position of infiltrates. The scale bar applicable to all micrographs = 200 μm. **(B)** Infiltrates were counted from Hoechst-stained cross-sections of lumbar spinal cord at Eo, Ep, and Ee. Bars represent mean number ± SEM, from *n* ≥ 6 sections per animal, from 3 animals per disease phase, from two independent experiments. Significance inside the graph: ^∗^*p* < 0.05 in respect to Eo. **(C)** Average number of cells within infiltrates. Bars present mean number ± SEM, counted from *n* ≥ 6 sections per animal, from 3 animals for each phase, from two independent experiments. Significance inside the graph: ^∗∗∗^*p* < 0.0001 in respect to Eo, ##*p* < 0.01 in respect to Ee. **(D–F)** Fractions of mononuclear infiltrates that express NTPDase1 at each phase of EAE were determined by flow cytometry. Representative dot-plots showing NTPDase1-immunoreactivity of mononuclear cells isolated from lumbar spinal cord. **(G)** Relative number of NTPDase1+ cells in mononuclear infiltrates during EAE, as determined by flow cytometry. Bars represent mean fraction (%) ± SEM of total NTPDAse1+ cells, obtained from 3 animals per group, from two independent experiments. Significance inside the graph: ^∗∗^*p* < 0.01, ^∗∗∗^*p* < 0.001 compared to Eo. **(B–G)** Kruskal–Wallis with Dunn’s *post hoc* test. Representative dot plots from flow cytometric analysis of mononuclear cells fraction at Ep, stained for NTPDase1 and CD4 **(H)** and for NTPDase1 and CD40 **(I)**. **(J)** Table represent results of flow cytometric analysis of cells stained for NTPDase1 and either CD4 or CD40, expressed as mean fraction (%) ± SEM. **(K,L)** Representative dot plot from flow cytometric analysis of mononuclear cells fraction at Ep, stained for NTPDase1 and CD45, with **(L)** showing boundaries between cells with low (R3), intermediate (R4), and high (R5) expression of CD45. **(M)** Table represent results of flow cytometric analysis of cells stained for NTPDase1 and CD40, expressed as mean fraction (%) ± SEM. **(J,M)** are from flow cytometric analysis performed on cells isolated at Ep from 10 animals.

The induction and progression of EAE were associated with progressive infiltration of peripheral immune cells (Figure 6A), that were observed in clusters of mononuclear cells, known to massively invade CNS during EAE. The number of infiltrates (Figure 6B) and a number of cells per infiltrate (Figure 6C) were low at ***Eo***, significantly increased at ***Ep*** and declined at ***Ee***, although the infiltrated cells were still apparent at the end of disease. The extent of cellular infiltrations in the lumbar spinal cord was evidenced at molecular level, by expression profiling of integrin αM (CD11b) gene (*Itgam*) and *Cx3cr1*, that reflects the presence of monocytes/macrophages and T cells, but also microglia, respectively. Eleven-fold increase in *Itgam*-mRNA and a fourfold increase in *Cx3cr1*-mRNA were demonstrated at ***Ep*** in respect to control (Table 3).

**Table 3:** Expression profiles for selected cellular markers of immune function.

Target gene	Target gene/GAPDH-mRNA (fold change in respect to control)		
	
	Eo	Ep	Ee
*Aif1*	5.2 ± 0.5**	14.5 ± 0.5***	4.5 ± 0.4*
*Nos2*	12 079 ± 2590***	1370 ± 389***	99 ± 39
*Arg1*	8.5 ± 0.7***	6.5 ± 1.7***	1.5 ± 0.2
*Cd68*	29.0 ± 5.4***	67.2 ± 3.8***	16.7 ± 2.4*
*Itgam*	11.3 ± 1.2***	11.0 ± 0.6***	3.9 ± 0.3
*Cx3cr1*	1.2 ± 0.2	4.1 ± 0.8**	3.1 ± 0.3*


**^∗^*p* < 0.05, ^∗∗^*p* < 0.01, ^∗∗∗^*p* < 0.001 in respect to control, Kruskal – Wallis with Dunn’s post hoc test.**

The contribution of mononuclear infiltrates to the overall increase in NTPDase1 expression in EAE was estimated by flow cytometry (Figure 6D–F). The cells were separated from the spinal cord tissue by differential centrifugation in Percoll gradient. The portion of NTPDase1+ cells in total mononuclear cell fraction increased from about 20% at ***Eo***, to about 70% at ***Ep*** (Figure 6G). The number of mononuclear cells decreased at Ee (Figure 6A–C), however, the fraction of NTPDase1+ cells as determined by flow cytometry, remained high, at about 60% (Figure 6G).

Since it was already shown that about half of the mononuclear cell fraction isolated at the peak of EAE represents T cells with prevalence of CD4+ subset, we assessed the contribution of CD4+ cells to NTPDase1 expression at the peak of EAE using flow cytometry (Figure 6H,J). In accordance with literature data, nearly 45% of mononuclears were CD4+ cells, with more than 70% of NTPDase1+ cells possibly indicating a regulatory subset of T cells. Our results also show that nearly half of NTPDase1+ mononuclear cell fraction belongs to CD4+ cells subset.

In addition, considering that co-stimulatory receptor CD40 represents a marker of activated microglia/macrophages, we estimated contribution of CD40+ cells to NTPDase1 expression in mononuclear fraction at Ep (Figure 6I,J). Our results show that CD40+ cells represent nearly 25% of total mononuclear fraction, with about 80% of NTPDase1+ cells. At the peak of EAE 40% of NTPDase1+ mononuclear cells were CD40+, indicating activated microglia/macrophages, but also dendritic cells and lymphocytes.

Finally, since mononuclear cell fraction at the peak of EAE is mostly comprised of CD45+ cells of haematopoietic origin including microglia/macrophages, we analyzed contribution of these cells to NTPDase1 expression at Ep by flow cytometry (Figure 6K–M). CD45+ cells comprised about 86% of mononuclear cells fraction, with almost 80% coexpressing NTPDase1. Also, among NTPDase1+ positive cells about 93% coexpressed CD45. Additionally, by setting boundaries between cell populations showing low, intermediate (int) and high (hi) CD45 expression, we evidenced about 29% of CD45^low^ (indicating resting microglia), 36% of CD45^int^ (indicating activated microglia) and 23% of CD45^hi^ (indicating macrophages and other haematopoietic cells such as dendritic cells or lymphocytes). NTPDase1 was expressed in about 62% of CD45^low^ fraction, 87% of CD45^int^ fraction, and 92% of CD45^hi^ fraction.

### Functional State of NTPDase1-ir Reactive Microglia/Macrophages

**FIGURE 7:**
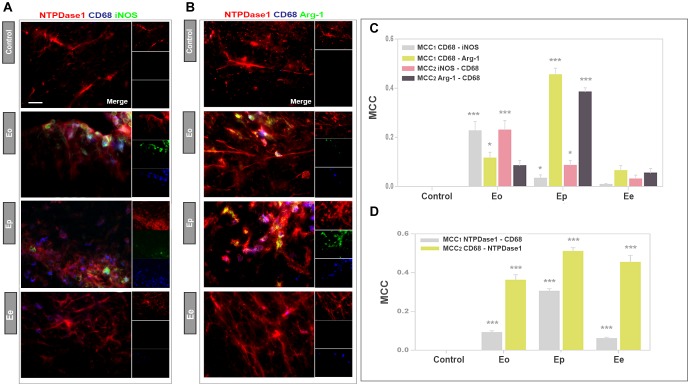
Functional state of NTPDase1 positive phagocytically active microglia/macrophages. **(A,B)** Representative micrographs showing triple immunofluorescence labeling directed to NTPDase1 (*red fluorescence*), CD68 (*blue fluorescence*), and iNOS or Arg1 (*green fluorescence*) at spinal cord cross-sections obtained from control animals and during EAE. Scale bar applicable to all micrographs = 20 μm. **(C,D)** MCC values obtained by multi-image colocalization analysis of micrographs represented in **(A,B)**, showing fractional overlap between pairs of signals, as indicated inside the graph. Bars represent mean value ± SEM, from *n* ≥ 4 images per spinal cord region, from *n* ≥ 6 sections per animal, from 3 animals per experimental group, from two separate experiments. Significance inside the graph: ^∗^*p* < 0.05; ^∗∗∗^*p* < 0.0001 in respect to control, Kruskal–Wallis with Dunn’s *post hoc* test.

The association of NTPDase1 with infiltrated monocytes/macrophages and reactive microglia during EAE was further assessed at spinal cord cross-sections, by double immunolabeling for NTPDase1 and CD68 (Figure 7), which marks the cells with phagocytic activity. The activation state of double NTPDase1/CD68stained cells was assessed by third immunofluorescent stain, directed either to iNOS (Figure 7A) or Arg1 (Figure 7B). As expected, control sections were completely devoid of CD68 reactivity. At Eo, CD68 immunoreactive cells with typical ovoid morphology appeared within infiltrates, mostly in subpial and perivascular region. At Ep, number of CD68 positive cells increased, while at Ee, only a few CD68-immunoreactive cells per section were observed. In all time-points, the majority of CD68 positive cells showed NTPDase1 immunoreactivity. CD68/iNOS double reactive cells were observed at Eo, while at Ep their number decreased. Regarding Arg1, only a few double CD68/Arg1 cells were observed at Eo, while at Ep their number increased. The micrographs were used for the determination of the MCC coefficients for co-occurrence between the corresponding signals. Microscopic observations were confirmed with calculated *MCC*_1_ coefficients for CD68/iNOS and CD68/Arg1 co-occurrence, respectively, indicating that only 5% of the total CD68 reactivity overlapped with iNOS, whereas almost 50% overlapped with Arg1 immunofluorescence at ***Ep*** (Figure 7C). Since values of *MCC*_2_ for NTPDase1/CD68 co-occurrence (Figure 7D) indicated that up to 50% of total CD68 fluorescence overlapped with NTPDase1 at ***Ep***, obtained results indicate that a significant portion of CD68 immunoreactive cells at the peak of EAE represent double NTPDase1/CD68 positive monocytes/macrophages and activated microglia that exhibit M2-like functional phenotype. In order to determine fractions of CD68 positive cells that coexpress either one or both activation state markers, we labeled consecutive sections for NTPDase1/CD68 and iNOS or Arg1 and counted cells at the overlayed micrographs (Supplementary Figure 3A). At Eo we observed a few CD68 positive cells coexpressing iNOS and Arg1, indicating mixed M1/M2 phenotype and a greater number of cells expressing only iNOS, indicating M1-like phenotype. In contrast, at Ep in increased number of CD68 reactive cells a few cells were iNOS/Arg1 positive while vast number of cells expressed only Arg1, indicating M2-like phenotype of microglia/macrophages. Quantification revealed that at Eo about 50% of CD68 positive cells coexpressed iNOS, while 20% were iNOS/Arg1 positive (Supplementary Figures 3B–E), indicating prevalence of M1-like phenotype. At Ep, Arg1 was expressed at 85%, while about 15% of CD68 reactive cells were iNOS/Arg1 positive, together implying prevalence of M2-like microglial/macrophages phenotype at the peak of EAE.

### Heterogeneity of NTPDase1 Immunoreactive Astrocytes

The preceding *in situ* hybridization/immunohistochemical study has demonstrated the presence of *Entpd1* mRNA in GFAP immunoreactive astrocytes mostly in the spinal cord white matter. Multi-image colocalization analysis has further revealed that the fraction of NTPDase1immunofluorescence that co-localizes with GFAP reactivity in spinal cord sections of control animals amounts to about 30% (Figure 4), and does not vary during EAE. However, given that GFAP reactivity only partially overlaps with NTPDase1, we explored the potential astrocyte heterogeneity based on the presence or absence of NTPDase1.

**FIGURE 8:**
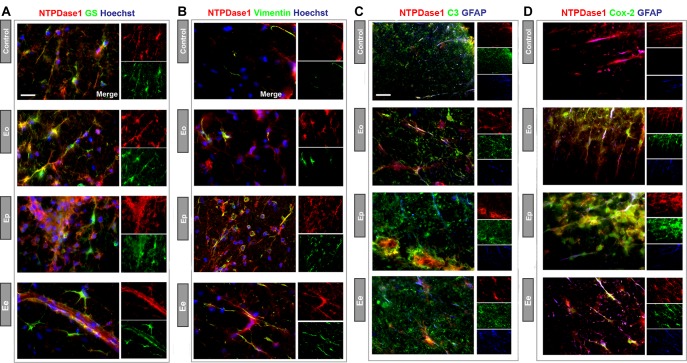
Heterogeneity of NTPDase1 positive astrocytes. Representative micrographs showing double immunofluorescence labeling directed to NTPDase1 (*red fluorescence*) and **(A)** glutamine synthetase – GS **(B)** vimentin, **(C)** Complement component 3 – C3, and **(D)** Cox-2 (*green fluorescence* for all). Sections **(A,B)** were counterstained with Hoechst for cell nuclei (*blue fluorescence*). Sections **(C,D)** were co-labeled with GFAP (*blue fluorescence*). Scale bar applicable to all micrographs = 20 μm.

The sections were double-immunolabeled for NTPDase1 and for either glutamine synthetase (Figure 8A), or vimentin (Figure 8B). The NTPDase1 fluorescence showed almost a complete overlap with both glutamine synthetase or vimentin immunoreactivity mostly in spinal cord white matter. During EAE, from Eo to Ee, glutamine synthetase positive astrocytes displayed shortening and thickening of processes and cell body hypertrophy indicating astrogliosis. Despite prominent labeling of gliotic astrocytes observed at Ee, Western blot analysis revealed no significant changes in glutamine synthetase protein expression (Supplementary Figure 4). Next, we have applied triple immunofluorescence labeling of NTPDase1/GFAP and either complement component 3 (Figure 8C) or inducible cyclooxygenase Cox2 (Figure 8D), which are distinctive markers of neurotoxic (A1) vs. neuroprotective (A2) astrocytes, respectively. As expected, the intensity of C3 immunoreactivity increased during EAE, mostly in infiltrated cells that co-expressed NTPDase1. With regard to astrocytes, although GFAP reactivity progressively increased during EAE, C3 fluorescence was barely seen in association with GFAP positive processes (Figure 8D). Cox2 immunoreactivity was undetectable in control sections. During EAE, Cox2 was strongly induced in astrocytes, while colocalization analysis confirmed increase in fraction of Cox2 fluorescence overlap with GFAP reactivity from Eo to Ee and stable overlap with NTPDase1 (Supplementary Figure 5), particularly in astrocytes in the white matter. The low C3- and strong Cox2-induction in astrocytes suggest generally low inflammatory potential of the tissue environment during EAE in accordance with our published results (Lavrnja et al., 2008, 2012) and suggest induction of beneficial phenotype of reactive astrocytes. Our preliminary results that show decreased P2Y_1_ receptor-immunoreactivity at Ep and Ee in fibrous processes in the white matter, indicating fibrous astrocytes (Supplementary Figure 6), speak in favor of the induction of the neuroprotective astrocyte phenotype in our EAE model.

### Expression of NTPDase1 by Neuronal Cells

**FIGURE 9:**
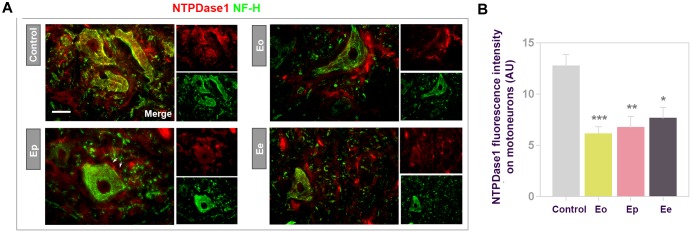
Expression of NTPDase1 by neuronal cells. **(A)** Representative micrographs showing double immunofluorescence labeling of spinal cord gray matter, with antibodies directed to NTPDase1 (*red fluorescence*) and neurofilament heavy subunit (NF-H) (*green fluorescence*). Arrows indicate microglial-like cells in close apposition with large motoneuron cell body. Scale bar applicable to all micrographs = 20 μm. **(B)** Integrated fluorescence density corresponding to NTPDase1 reactivity associated with large motoneuron cell body. The intensity was determined as signal confined within an outline of motoneuron cell body, from at least 3 neurons per section, from *n* ≥ 6 sections per animal, from 3 animals per experimental group. Significance inside the graph: ^∗^*p* < 0.01, ^∗∗^*p* < 0.001, and ^∗∗∗^*p* < 0.0001, one – way ANOVA with Dunnet’s *post hoc* test.

Considering that *in situ* hybridization/immunohistochemistry revealed a subpopulation of large motoneurons in ventral gray matter expressing *Entpd1* mRNA, we further investigated NTPDase1 expression during EAE in these cells by double immunofluorescence histochemistry directed to NTPDase1 and neurofilament heavy subunit NF-H (Figure 9A). In control sections, NTPDase1immunoreactivity was mostly present at large motoneurons in ventral gray matter and the overall NTPDase1 fluorescence intensity decreased during EAE. Quantification of NTPDase1 fluorescence intensity corresponding to individual neuronal cell bodies in ventral gray matter showed significant down-regulation evidenced until the end of the disease (Figure 9B). At Eo and Ep, neuronal cell bodies were seen in close apposition with NTPDase1-positive ovoid cells.

## Discussion

The aim of the present study was to identify cell types that are responsible for massive up-regulation of NTPDase1 in the spinal cord of animals affected by EAE, and the potential pathophysiological significance of such changes. Briefly, the expression of *Entpd1* mRNA and NTPDase1 protein increased with the onset of symptoms, and reached a maximum at the peak of the disease, being about 3- and 2-fold higher than in control, respectively. At the end of the disease, NTPDase1 mRNA and protein expression returned to the control levels. *In situ* hybridization and immunofluorescence labeling of NTPDase1, combined with a second label directed toward the selective cellular markers of neurons, microglia/macrophages or astrocytes, were used to establish the cellular contribution of strongly enhanced NTPDase1 during EAE. In accordance with the existing literature data (Braun et al., 2000), in control animals, NTPDase1 mostly resided at ramified microglia and vascular endothelium, while prominent labeling was found at large neuronal cell bodies in the ventral gray matter, and at fibrous astrocytes in the white matter. However, during EAE, activated microglia and mononuclear infiltrates accounted for most of the enhanced NTPDase1 expression, whereas the expression levels of NTPDase1 even slightly decreased at neurons and astrocytes. Given that reactive microglia/macrophages and astrocytes are beside CD4+T cells the most important effectors of neuroinflammation during EAE (Murphy et al., 2010; Brambilla et al., 2014), we next assessed individual contribution of each of these cell types to observed increase in NTPDase1 expression along with the functional state of these cells during EAE.

Double-immunofluorescence labeling and co-localization analysis revealed that in control sections, about 40% of total NTPDase1 immunoreactivity resides at Iba1-positive cells, while up to 80% of Iba1 immunoreactivity overlapped with NTPDase1 fluorescence, which is in accordance with our microscopic observations and literature data (Braun et al., 2000) on constitutive expression of NTPDase1 by quiescent microglia. The abundance of Iba1, which tags microglia/macrophages irrespective of their functional state, increased several-fold during EAE, reaching the maximum at ***Ep***, due to the increased number of activated microglial cells and macrophages attracted by neuroinflammation. However, despite strong Iba1 up-regulation, the fractional overlap between Iba1- and NTPDase1-immunofluorescence remained steadily at 80%. Such high degree of fractional overlap point to microglia/macrophages as cells accountable for the most of the enhanced NTPDase1 expression during EAE. These results are in compliance with previously reported up-regulation of NTPDase1 at microglia in neuroinflammatory conditions induced by traumatic brain injury (Nedeljkovic et al., 2006; Simon et al., 2017). Furthermore, stable high levels of Iba1/NTPDase1 co-occurrence confirmed our observations that NTPDase1 is expressed by the vast majority of microglia/macrophages, irrespective of their activation state.

We evidenced a massive infiltration of mononuclear cells in the spinal cord tissue at ***Ep*** using immunohistochemistry and by detecting several-fold induction of *Itgam* and *Cx3cr1* mRNA (Sunnemark et al., 2005; Greter et al., 2015). The contribution of infiltrated mononuclear cells to the observed up-regulation of NTPDase1 was assessed by flow cytometry. The fraction of infiltrated NTPDase+ cells in general mononuclear population increased from about 20% at ***Eo***, to about 80% at ***Ep***. It was previously shown that microglia and macrophages account for 12% of mononuclear fraction isolated from spinal cord at the peak of disease, while T cells constitute about 40% (White et al., 1998), out of which the majority are CD4+ subset that is crucial in neuroinflammation during EAE (Dittel, 2008; Murphy et al., 2010; Constantinescu et al., 2011). In accordance with previously reported data from the same EAE model (Miljkovic et al., 2011), in mononuclear fraction isolated from the spinal cord at the peak of EAE was evidenced almost 45% of CD4+ cells, mostly comprising T lymphocytes. About 70% of these cells were NTPDase1/CD39+, possibly indicating regulatory subset (Borsellino et al., 2007). This means that at Ep about 30% of mononuclear fraction, otherwise maximal at that time-point, probably belongs to Tregs subset. It was previously shown that Tregs crucially contribute to spontaneous resolution of neuroinflammation in EAE/MS (Kleinewietfeld and Hafler, 2014; Koutrolos et al., 2014; Wang et al., 2014). Additionally, constitutive expression of both NTPDase1/CD39 and e-5NT/CD73 was reported in murine Tregs (Borsellino et al., 2007; Deaglio et al., 2007). Considering our results, through secretion of antiinflammatory cytokines (Koutrolos et al., 2014) and ATP removal coupled with adenosine production (Deaglio et al., 2007), Tregs might sustain antiinflammatory transition of microglia/macrophages at the peak of neuroinflammation.

It was previously shown that co-stimulatory receptor CD40, which is critical for reactivation of autoaggressive T cells (Becher et al., 2001), has a key role in neuroinflammation (Aarts et al., 2019). Additionally, it was reported that CD40 was expressed in approximately 45% of activated microglia and 73% of macrophages in mononuclear cells fraction at the peak of EAE (Aarts et al., 2017). Accordingly, our results showed that nearly 30% of isolated mononuclears at Ep were CD40+ cells, out of which 82% coexpressed NTPDase1. Although in microglia/macrophages CD40 is regarded as M1 marker (Aarts et al., 2017), it was evidenced that about 70% of CD40 positive microglia/macrophages in MS lesions coexpress markers of M2 phenotype, indicating frequent CD40 association with mixed M1/M2 phenotype (Vogel et al., 2013). This also suggests that at least part of CD40/NTPDase1 positive microglia/macrophages evidenced at Ep in our EAE model may exhibit some form of intermediary M1/M2 phenotype.

In accordance with literature data (White et al., 1998) mononuclear fraction isolated at the peak of EAE comprised nearly 87% of CD45+ cells, indicating both blood-borne cells and resident microglial cells, abundantly expressing NTPDase1. On the basis of relative CD45 expression (Zhang et al., 2002; Rumble et al., 2015) we evidenced about 29% of CD45 low-expressing cells, indicative of resting microglia, 36% of intermediary-expressing indicating mostly activated microglia and 24% of CD45 high-expressing mononuclear cells comprising macrophages and other heamatopoetic cells, mostly in compliance with literature data (Miljkovic et al., 2011; Rumble et al., 2015; Stojić-Vukanić et al., 2016). Increase in fraction of CD45/NTPDase1 positive cells from low- to high expressing CD45+ cells imply that activation induced by neuroinflammation enhances NTPDase1 expression in microglia/macrophages and also indicates largest contribution of activated microglia to increase in total NTPDase1 expression in spinal cord at the peak of EAE.

We also determined the expression of NTPDase1 at a subpopulation of phagocytic cells, which were identified by the presence of CD68 antigen. At the onset of EAE, about 10% of -total NTPDase1-immunoreactivity resided at CD68 positive cells. The expression of CD68 increased several-fold during EAE, apparently due to an increased number of phagocytic microglia/macrophages (Supplementary Figure 3B) in the neuroinflammatory disease (Fu et al., 2014), while the fraction of NTPDase1 immunofluorescence overlapped with CD68 reactivity increased to about 30% at Ep. Taken together, our findings suggest that phagocytically active cells are accountable for about 30% of increased NTPDase1 expression at the peak of EAE.

Under neuroinflammatory conditions, quiescent microglia and peripheral macrophages undergo conspicuous morphological changes, along which they acquire functional characteristics of reactive cells (Volonte et al., 2016). The reactive microglia/macrophages may acquire any phenotype within the spectrum between two extremes, termed M1 and M2 (Fumagalli et al., 2015; Kalkman and Feuerbach, 2016; Parisi et al., 2016; Geloso et al., 2017). It was established *in vitro* that classically activated M1 microglia/macrophages, induced by LPS and IFNγ, exhibit cytotoxic and pro-inflammatory actions, while alternatively, IL-4 activated M2 microglia/macrophages, exhibit protective and anti-inflammatory properties (Porta et al., 2009; Kalkman and Feuerbach, 2016). Since peripheral macrophages *in vitro* down-regulate NTPDase1 during the polarization toward the M1 phenotype (Zanin et al., 2012), we have hypothesized that the transition from M1 to M2 phenotype in microglia and infiltrated macrophages during the acute neuroinflammation in EAE, might be associated with the up-regulation of NTPDase1 by those cells. Given that the most commonly used differential markers of M1 and M2 functional states are iNOS and Arg1, respectively (Cherry et al., 2014), we applied multi-immunofluorescence labeling to test this hypothesis.

The acute neuroinflammatory response in EAE was associated with a biphasic pattern of expression of several reactive microglia and astrocytic markers, including iNOS and Arg1. Expression of both reactive markers massively increased already at ***Eo***, and gradually decreased thereafter, returning to the control level at ***Ee***. On the other hand, iNOS immunoreactivity was increased at the onset and peak, while Arg1 fluorescence was most prominently upregulated at the peak of EAE. However, while the highest overlap between Iba1- and iNOS-immunoreactivity occurred at ***Eo***, the highest overlap between Iba1/Arg1-immunoreactivity occurred at ***Ep***, suggesting a prevalence of M1-like reactive microglia/macrophages at the onset and the M2-like phenotype at the peak of EAE. Additionally, in accordance with literature data showing that activated microglia/macrophages *in vivo* frequently express mixed markers of M1 and M2 phenotype (Fumagalli et al., 2015; Kalkman and Feuerbach, 2016), we observed double iNOS/Arg1 reactivity in one part of Iba1 positive cells at Eo, and much less at Ep. Prevalence of iNOS positive cells at Eo and Arg1 positive cells at Ep over double iNOS/Arg1 reactive microglia/macrophages additionally indicates transition to M2-like phenotype at the peak of neuroinflammation. Similarly to Iba1/iNOS and Iba1/Arg1 co-occurence, about 10% of NTPDase1-fluorescence overlapped with iNOS-reactivity at ***Eo***, while the highest co-occurrence of NTPDase1- with Arg1-reactivity of about 30% was found at ***Ep***, again suggesting a higher association of NTPDase1 with M2-like microglia/macrophages.

In a similar fashion, analysis of iNOS and Arg1 expression in the phagocytically active cells expressing CD68, revealed the transition from the M1-like state at ***Eo*** to prevalent M2-like state in ***Ep***. Namely, colocalization analysis revealed about two-fold higher colocalization of CD68 immunofluorescence with iNOS compared to Arg1 reactivity at the onset, while at the peak about half of otherwise increased CD68 immunofluorescence colocalized with Arg1 as opposed to almost negligible colocalization with iNOS.

Regarding cellular expression, at the onset iNOS expressing CD68 positive cells, indicating M1-like activation state, prevailed over cells exhibiting mixed phenotype, while at the peak, Arg1-expressing cells were prevalent, additionally confirming transition to antiinflammatory activation state in microglia/macrophages at the peak of EAE.

Apart from T and B lymphocytes, microglia and macrophages are the predominant immune cell types in inflammatory demyelinating lesions in MS/EAE (Lassmann, 2018) and their functional phenotype directs the course of neuroinflammation toward progression or resolution. The transition between the reactive states is associated with a transcriptional shift in several thousand genes, including the genes coding the enzymes involved in arginine metabolism (Xu et al., 2003). Namely, the M1 vs. M2 microglia/macrophage classification can be condensed into two opposing pathways for arginine metabolism, mediated via iNOS or via Arg1, respectively. Both enzymes compete for L-arginine and metabolize it to NO and citrulline or ornithine and urea, with opposite effects regarding neuroinflammation (Morris, 2007; Fenn et al., 2014). The M1/M2 transition is associated with a significant shift in cytokine production (Franco and Fernandez-Suarez, 2015), which was also reported in our previous papers (Lavrnja et al., 2008, 2012), that directs T lymphocytes to produce Th1 or Th2 responses, that further amplify M1 or M2 type responses in positive feedback loops stabilizing the predominant immune phenotype.

In our study, the transition of microglia/macrophages from M1- to M2-like reactive state occurred at ***Ep*** in a significant fraction of about 80% of otherwise significantly enhanced activated cells, evidenced at the peak of symptoms, the majority of which co-expressed NTPDase1. Thus, our results indicate a strong association of NTPDase1 with antiinflammatory microglial phenotype and potentiation of its immunosuppressive effects (Pesce et al., 2009).

Another functional consequence of prevalent M2-like microglia/macrophage state at ***Ep*** may be the influence on functional state of astrocytes, since reactive microglia acts as the major inducer of reactive astrocyte phenotype in neuroinflammatory conditions (Liddelow et al., 2017). Thus, prevailing antiinflammatory microglial/macophages phenotype may propel reactive astrocytes toward the beneficial, neurotrophic phenotype. Also, enhanced expression of NTPDase1 by microglia/macrophages demonstrated in our EAE model, may potentiate development of neuroprotective phenotype in astrocytes through decreased P2Y_1_ signaling (Shinozaki et al., 2017). Additionally, we have previously evidenced decrease in mRNA and protein expression of P2Y_1_ during EAE (Jakovljevic et al., 2017) while our preliminary results (shown in Supplementary Figure 6) point to fibrous astrocytes as holders of the observed changes, implying induction of neuroprotective phenotype in astrocytes (Shinozaki et al., 2017). It was shown that reactive neurotrophic A2 astrocytes up-regulate many factors which support the survival and growth of neurons (Zamanian et al., 2012; Liddelow et al., 2017). Cyclooxygenase 2 (Cox2) is among highly induced and most specific genes in beneficial astrocytes phenotype, with strong protective effects in neuroinflammation (Aïd and Bosetti, 2011). We have evidenced a prominent induction of Cox2 in astrocytes during EAE, additionally increasing from the onset toward the resolution phase. Additionally, Cox2 was distinctively colocalized with NTPDase1 at astrocytes, particularly in the white matter, indicating their protective phenotype. Although results of Western blot analysis did not show changes in glutamine synthetase protein abundance during EAE, we observed strong labeling of hypertrophied bodies and thickened processes of perivascular astrocytes and their endfeet at Ee, indicating intense glutamate and ammonia removal at gliovascular unit. The up-regulation of Cox2 may be involved in a modulation of cerebral blood flow by perivascular astrocytes in response to neuroinflammation (Font-Nieves et al., 2012; Macvicar and Newman, 2015), which together with glutamine synthetase may support the recovery of the blood-brain barrier function in EAE (Bennett et al., 2010).

What is the physiological significance of NTPDase1 induction by reactive microglia/macrophages during EAE? It is widely accepted that ATP and adenosine arise in the extracellular space in response to any type of brain insult to initiate and direct response of microglia and astrocytes (Rodrigues et al., 2015). Thus, NTPDase1, 2 and e-5NT calibrate duration, magnitude, and composition of the “purinergic halo” surrounding the resident glial and infiltrated immune cells. We have already reported dynamic changes in the expression and activity of NTPDase2 and e-5NT by reactive astrocytes, and the expression of the whole set of P1 and P2 receptors embedded in the pathophysiological context associated with EAE (Lavrnja et al., 2009, 2015; Jakovljevic et al., 2017). Briefly, the acute course of EAE is associated with a significant decrease in ADP-producing NTPDase2 activity and strong attenuation of ADP-sensitive P2Y_1_, P2Y_12,_ and P2Y_13_ signaling at the peak of disease (Jakovljevic et al., 2017). On the other hand, the resolution of the acute neuroinflammation in EAE is associated with massive induction of NTPDase1 [(Jakovljevic et al., 2017), present paper] and e-5NT (Lavrnja et al., 2015), and attenuation of P2X and potentiation of A_2A_ and A_2B_ receptor signaling (Lavrnja et al., 2015; Jakovljevic et al., 2017). NTPDase1 and e-5NT, strongly induced in EAE by reactive microglia/macrophages and astrocytes, respectively, expedite degradation of pro-inflammatory ATP and production of anti-inflammatory adenosine, which is known to modulate the course and dictate the outcome of several neuroinflammatory conditions (Gomes et al., 2011), including EAE. Given that NTPDase1/e-5NT tandem is able to effectuate the whole sequence of extracellular ATP degradation, they may be viewed as an “immunological switch” that shifts pro-inflammatory immune cell activity toward the anti-inflammatory state (Antonioli et al., 2013). Therefore, by regulating ATP/adenosine ratio in the extracellular space, NTPDase1/e-5NT axis participates in the fine-tuning of microglial/macrophage and astrocyte differentiation and activity, as it has been already established for peripheral macrophages. Specifically, deficiency of NTPDase1 in peripheral macrophages results in ATP accumulation and their polarization toward the M1-like phenotype characterized by the release of proinflammatory cytokines IL-1β, IL-18, IL-6, and TNF-α (Zanin et al., 2012). Accordingly, the enhanced expression and activity of both NTPDase1/e-5NT in M2 macrophages alters ATP/adenosine ratio and inflammatory cytokine release. Thus, it is likely that highly up-regulated NTPDase1 at the peak of EAE, represents a contribution to or reflection of enhanced production of adenosine, and transition from M1- to M2-like microglia/macrophages phenotype. Since in human MS, M2 microglial/macrophages polarization arises from Th2 immune responses, and it further influences the balance between Th1/Th17 and Treg cells, there has been a strong rationale for therapies in MS that favor M2 differentiation (Liu et al., 2013). Our study provides new data regarding the involvement of ectonucleotidases in molecular events underlying the development of M2-like microglia/macrophages phenotype, which may represent the basis for M2 potentiating therapeutic strategies based on a modulation of purinergic signaling.

## Ethics Statement

Experimental protocols were approved by the Ethical Committee for the Use of Laboratory Animals of the Institute of Biological Research “Siniša Stanković,” Belgrade, Serbia (Application No. 01-11/14) and in compliance with the ECC Directive (2010/63/EU) on the protection of animals used for experimental and other scientific purposes.

## Author Contributions

All listed authors contributed to various aspects of the work, such as experimental design, acquisition, analysis and interpretation of data, finally approved submitted version of the manuscript and agreed to be amenable for all aspect of the work. DL, MJ, IL, and NN conceived and designed the experiments. MJ, DL, IBo, IL, AM, IBj, DS, and SP performed the experiments. DL, NN, MJ, and IL analyzed the data. DL, NN, IL, MJ, and JS contributed to the writing of the manuscript.

## Conflict of Interest Statement

The authors declare that the research was conducted in the absence of any commercial or financial relationships that could be construed as a potential conflict of interest.

## Footnotes

1 http://imagej.nih.gov/ij/download.html
