# Supplementary material for: Induction of NTPDase1/CD39 by Reactive Microglia and Macrophages Is Associated With the Functional State During EAE
Source: Front Neurosci. 2019 Apr 26;13:410. doi: 10.3389/fnins.2019.00410 (PMC6498900; doi:10.3389/fnins.2019.00410)
Supplement: Supplementary file 1 [file Data_Sheet_1.pdf]

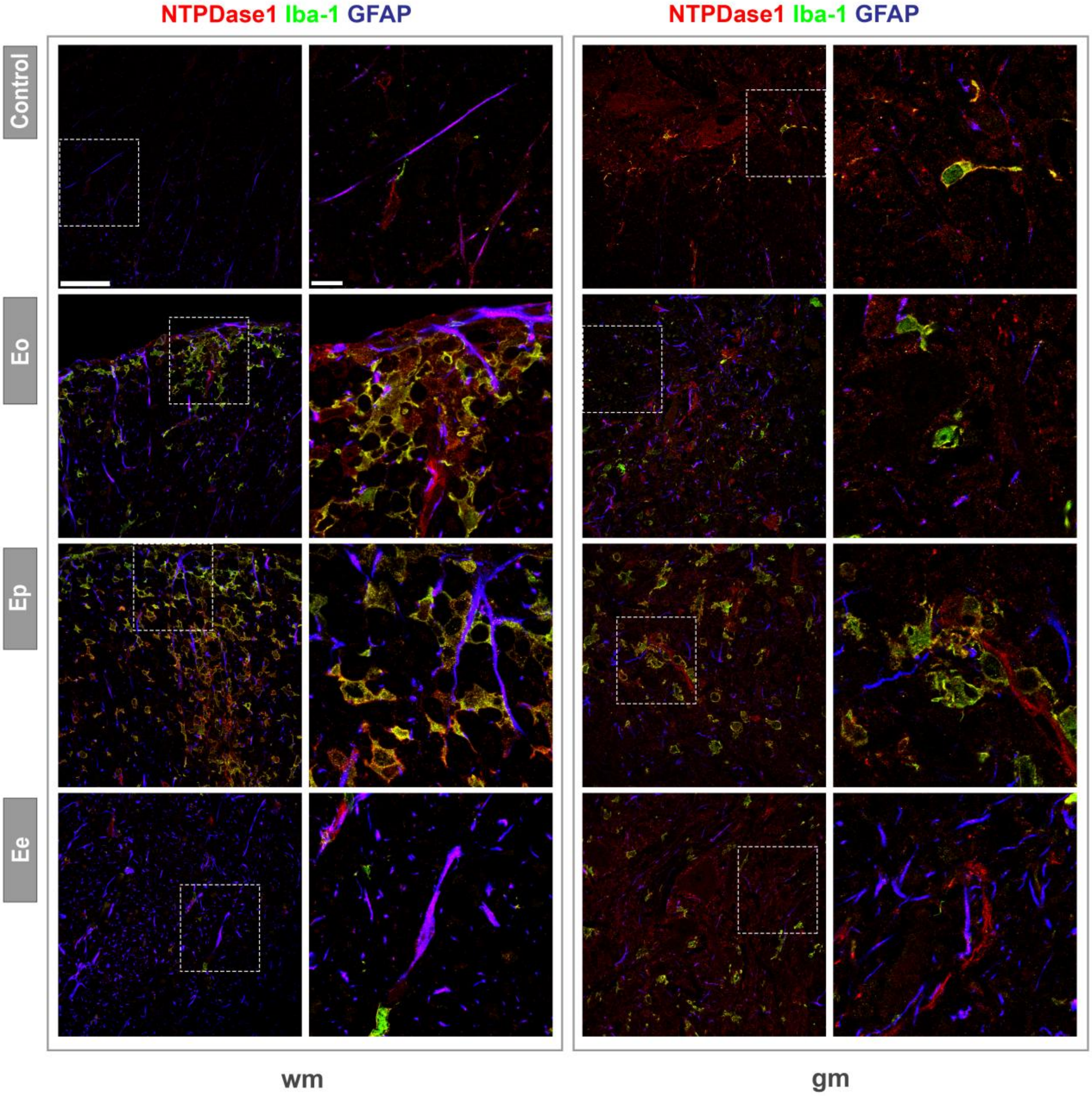

	Control	Eo	Ep	Ee
Overlap coefficient Iba-1 - NTPDase1	0.59±0.07	0.69±0.04	0.76±0.05	0.58±0.01
Overlap coefficient GFAP - NTPDase1	0.52±0.07	0.49±0.05	0.4±0.02	0.56±0.11

Supplementary Fig. 1. Expression of NTPDase1 by microglia/macrophages and astrocytes analyzed by confocal microscopy. Representative low-power (scale bar=50μm) and high-power (scale bar=10μm) micrographs of spinal cord cross-sections from white matter (wm) and gray matter (gm), respectively, showing triple immunofluorescence labeling directed to

NTPDase1 (*red fluorescence*), Iba1 (*green fluorescence*) and GFAP (*blue fluorescence*). Micrographs from control and EAE animals were obtained by Leica confocal microscope TCS MP5. The degree of GFAP/NTPDase1 and Iba-1/NTPDase1 overlap were analyzed by Leica Microsystems LAS AF TCS MP5 Software®. Overlap coefficients presented as mean ± SEM are shown in the table.
