# Supplementary material for: Induction of NTPDase1/CD39 by Reactive Microglia and Macrophages Is Associated With the Functional State During EAE
Source: Front Neurosci. 2019 Apr 26;13:410. doi: 10.3389/fnins.2019.00410 (PMC6498900; doi:10.3389/fnins.2019.00410)
Supplement: Supplementary file 2 [file Data_Sheet_2.pdf]

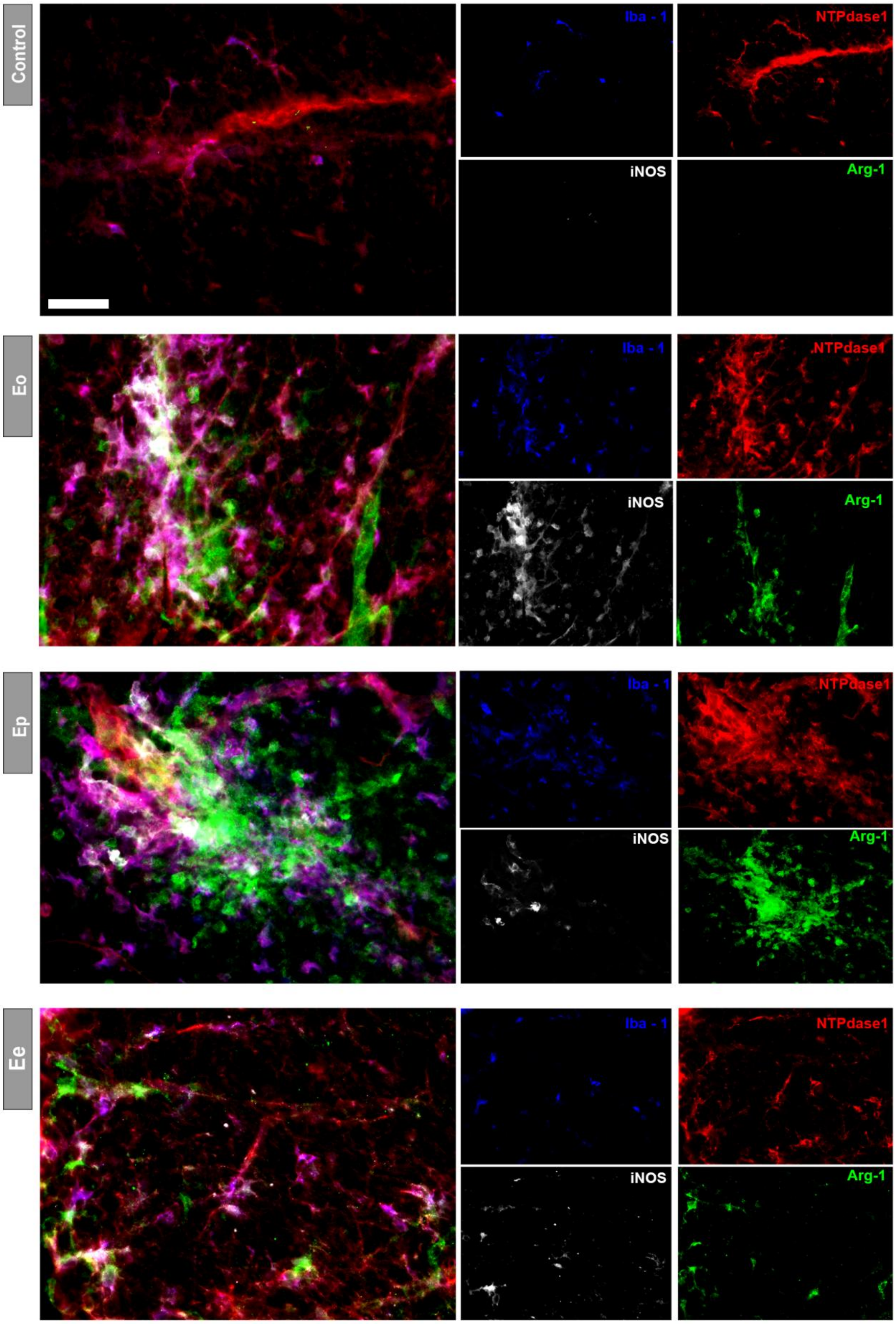
Control
Iba - 1
NTPdase1
iNOS
Arg-1
Eo
Iba - 1
NTPdase1
iNOS
Arg-1
Ep
Iba - 1
NTPdase1
iNOS
Arg-1
Ee
Iba - 1
NTPdase1
iNOS
Arg-1

Supplementary Fig. 2. Functional state of reactive microglia/macrophages. Representative micrographs showing immunofluorescence labeling directed to NTPDase1 (*red fluorescence*), Iba1 (*blue fluorescence*), iNOS (*white* fluorescence) and Arg1 (*green fluorescence*). Micrographs are obtained by overlay of triple NTPDase1/Iba1/iNOS with Arg1 fluorescence of consecutive spinal cord cross-sections obtained from control animals and during EAE. Scale bar applicable to all micrographs = 20 μm.
