# Supplementary material for: Induction of NTPDase1/CD39 by Reactive Microglia and Macrophages Is Associated With the Functional State During EAE
Source: Front Neurosci. 2019 Apr 26;13:410. doi: 10.3389/fnins.2019.00410 (PMC6498900; doi:10.3389/fnins.2019.00410)
Supplement: Supplementary file 3 [file Data_Sheet_3.pdf]

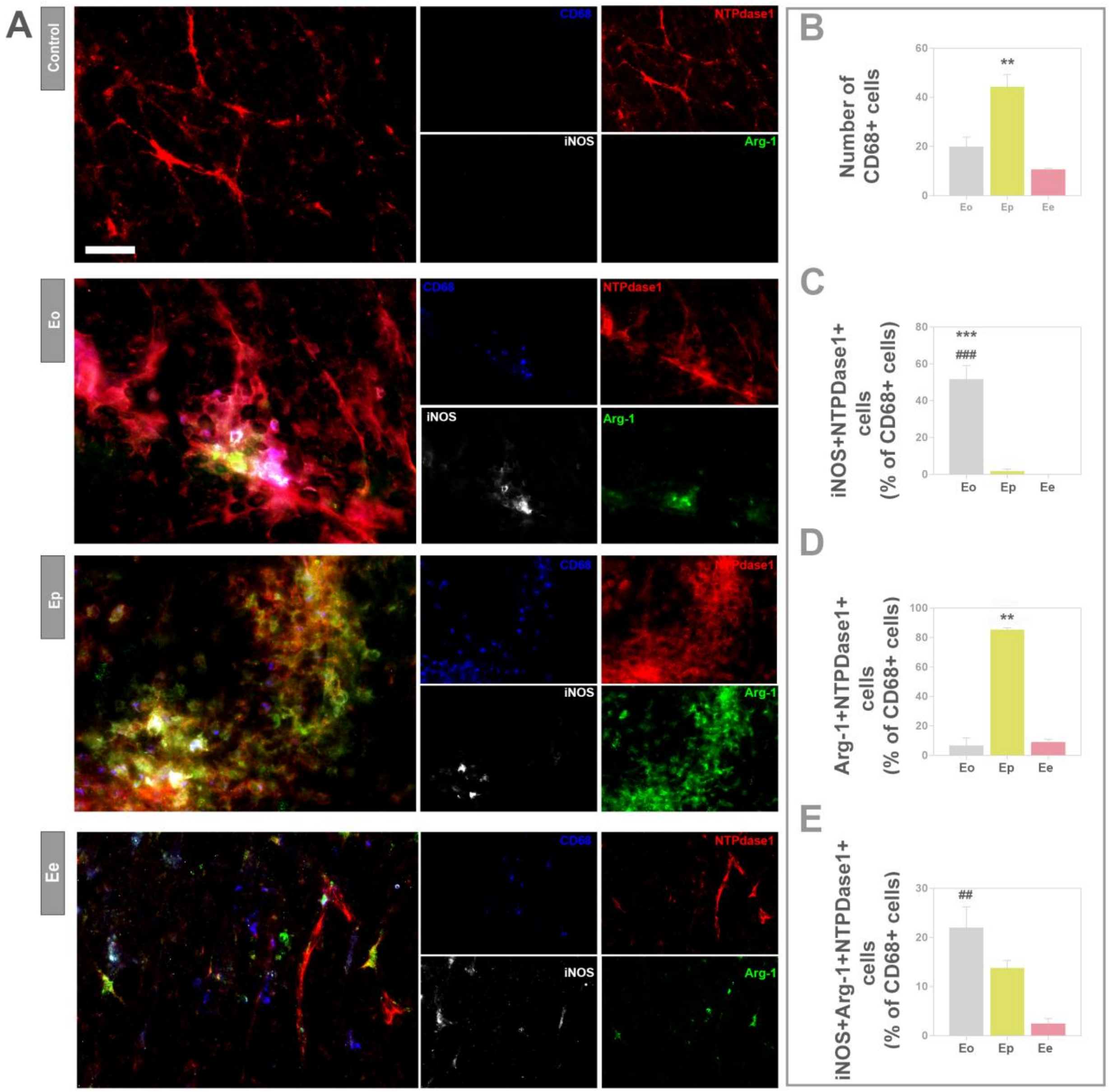

Supplementary Fig. 3. Functional state of NTPDase1 positive phagocytically active microglia/macrophages. Representative micrographs showing immunofluorescence labeling directed to NTPDase1 (*red fluorescence*), CD68 (*blue fluorescence*), iNOS (*white* fluorescence) and Arg1 (*green fluorescence*). Micrographs are obtained by overlay of triple NTPDase1/CD68/iNOS with Arg1 fluorescence of consecutive cross-sections obtained from control animals and during EAE (A). Scale bar applicable to all micrographs = 20 µm. Quantification graphs showing number of CD68 positive cells per micrograph (B), fraction of iNOS/NTPDase1 (C), Arg1/NTPDase1 (D) and iNOS/Arg1/NTPDase1 (E) positive cells represented as fraction (%) ± SEM of total CD68 positive cells. Significance inside the graphs: $^{**}p<0.01$ between Ep and Ee in (B); $^{***}p<0.001$ between Eo and Ep and $^{###}p<0.001$ between Eo

and Ee in (C); **$p<0.01$ between Eo and Ep in (D) and $^{\#\#}p<0.01$ between Eo and Ee in (E), Kruskal – Wallis with Dunn’s posthoc test.
