# Supplementary material for: Induction of NTPDase1/CD39 by Reactive Microglia and Macrophages Is Associated With the Functional State During EAE
Source: Front Neurosci. 2019 Apr 26;13:410. doi: 10.3389/fnins.2019.00410 (PMC6498900; doi:10.3389/fnins.2019.00410)
Supplement: Supplementary file 4 [file Data_Sheet_4.pdf]

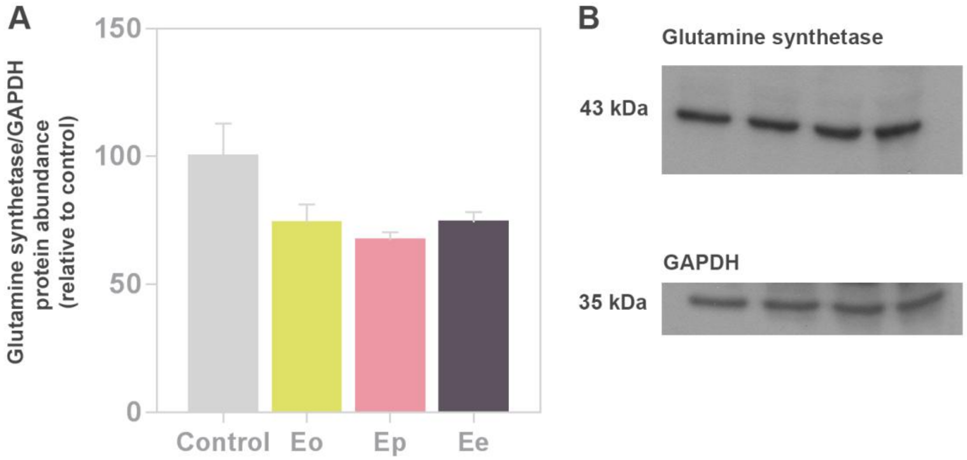

Supplementary Figure 4. Glutamine synthetase protein abundance during the course of EAE. (A) Glutamine synthetase/GAPDH value obtained for the control sample was defined as 100% ± SEM and the ratios obtained for other samples were expressed relative to the control (bars). Bars represent the mean glutamine synthetase protein abundance (±SEM) from $n \geq 2$ determinations. (B) Representative immunoblots for glutamine synthetase and GAPDH as a loading control.
