# Supplementary material for: Induction of NTPDase1/CD39 by Reactive Microglia and Macrophages Is Associated With the Functional State During EAE
Source: Front Neurosci. 2019 Apr 26;13:410. doi: 10.3389/fnins.2019.00410 (PMC6498900; doi:10.3389/fnins.2019.00410)
Supplement: Supplementary file 5 [file Data_Sheet_5.pdf]

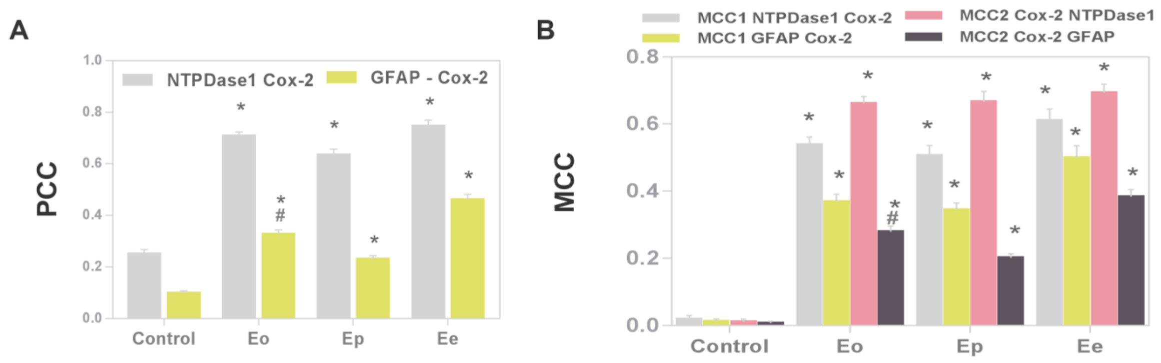

Supplementary Figure 5. Quantification of colocalization of Cox2, NTPDase1 and GFAP immunofluorescence during the course of EAE. Quantification graph (A): Pearson's correlation coefficients (PCC), showing level of overlap between NTPDase1/Cox2 immunofluorescence (gray bars) and GFAP/Cox2 immunofluorescence (yellow bars) in the white and gray matter of spinal cord cross-sections Bars represent mean PCC ± SEM from $n \geq 4$ micrographs per section, for $n \geq 6$ sections per animal, from 3 animals per experimental group, from two separate experiments. Manders' colocalization coefficients MCC_1 and MCC_2 (B), showing fractional overlap between pairs of signals as shown inside the graph, obtained for the whole spinal cord cross-section. Bars represent mean ± SEM from $n \geq 4$ micrographs per section, $n \geq 6$ sections per animal, from 3 animals per experimental group, from two separate experiments.. Significance inside the graphs: $^{*}p < 0.0001$ in comparison to control, $^{\#}p < 0.05$ in comparison to Ee, Kruskal – Wallis with Dunn's posthoc test.
