# Supplementary material for: Induction of NTPDase1/CD39 by Reactive Microglia and Macrophages Is Associated With the Functional State During EAE
Source: Front Neurosci. 2019 Apr 26;13:410. doi: 10.3389/fnins.2019.00410 (PMC6498900; doi:10.3389/fnins.2019.00410)
Supplement: Supplementary file 6 [file Data_Sheet_6.pdf]

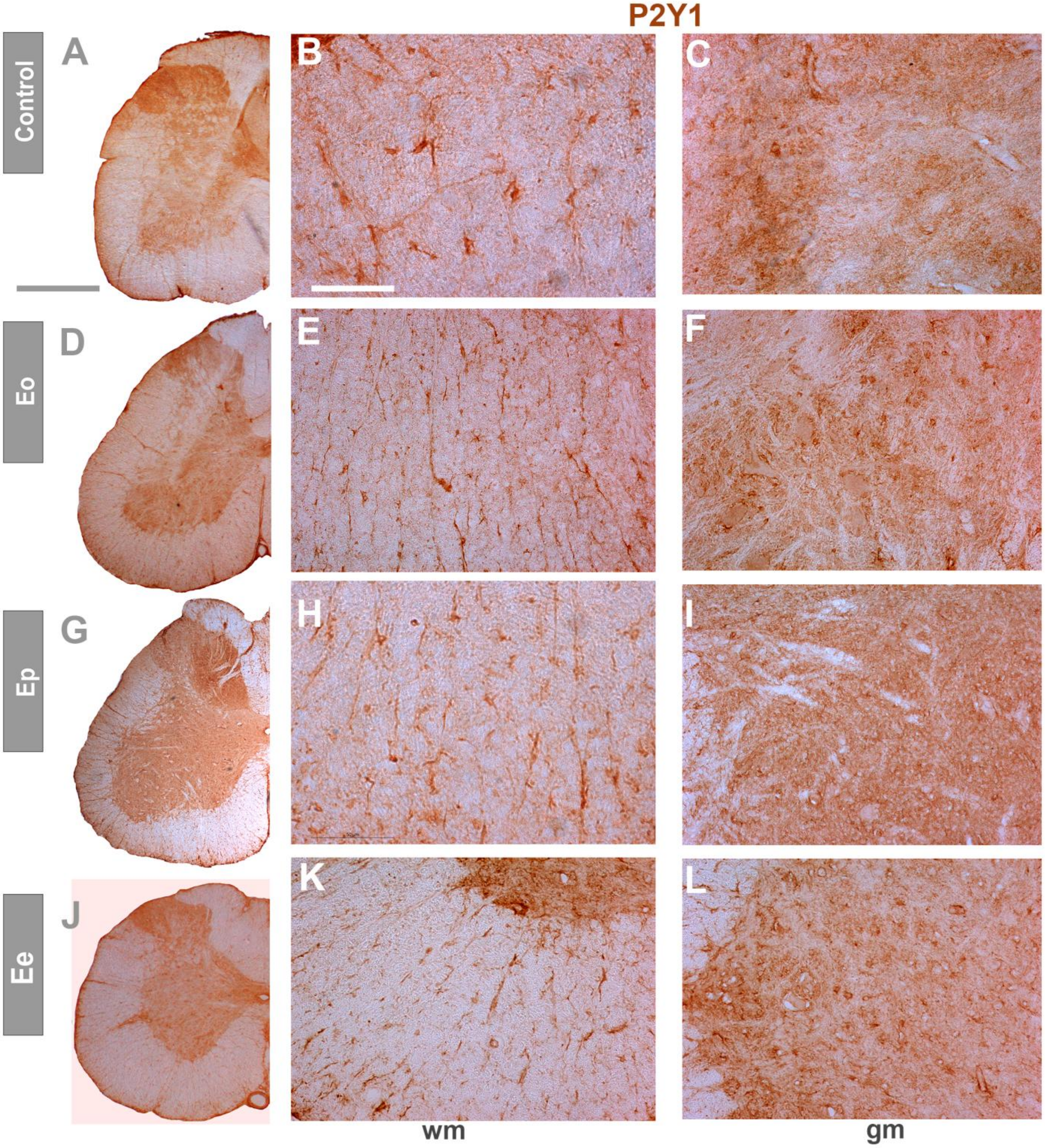

Supplementary Fig. 6. Immunohistochemical localization of $P2Y_1$ receptor in lumbosacral cross-sections obtained in control animals and during EAE. Low-power magnification micrographs showing the distribution of $P2Y_1$ immunoreactive elements in control (A) and EAE (D, G, J) spinal cord cross-sections. High-power magnification showing the distribution of $P2Y_1$ immunoreactive elements in white (B, E, H, K) and grey matter (C, F, I, L) of spinal cord cross-sections obtained from control animals and during EAE. Scale bar at (A) applicable to (D, G, J) = 500 μm. Scale bar at (B) applicable to (E, H, K, C, F, I, L) = 50 μm.
