# Supplementary material for: Induction of NTPDase1/CD39 by Reactive Microglia and Macrophages Is Associated With the Functional State During EAE
Source: Front Neurosci. 2019 Apr 26;13:410. doi: 10.3389/fnins.2019.00410 (PMC6498900; doi:10.3389/fnins.2019.00410)
Supplement: Supplementary file 7 [file Data_Sheet_7.pdf]

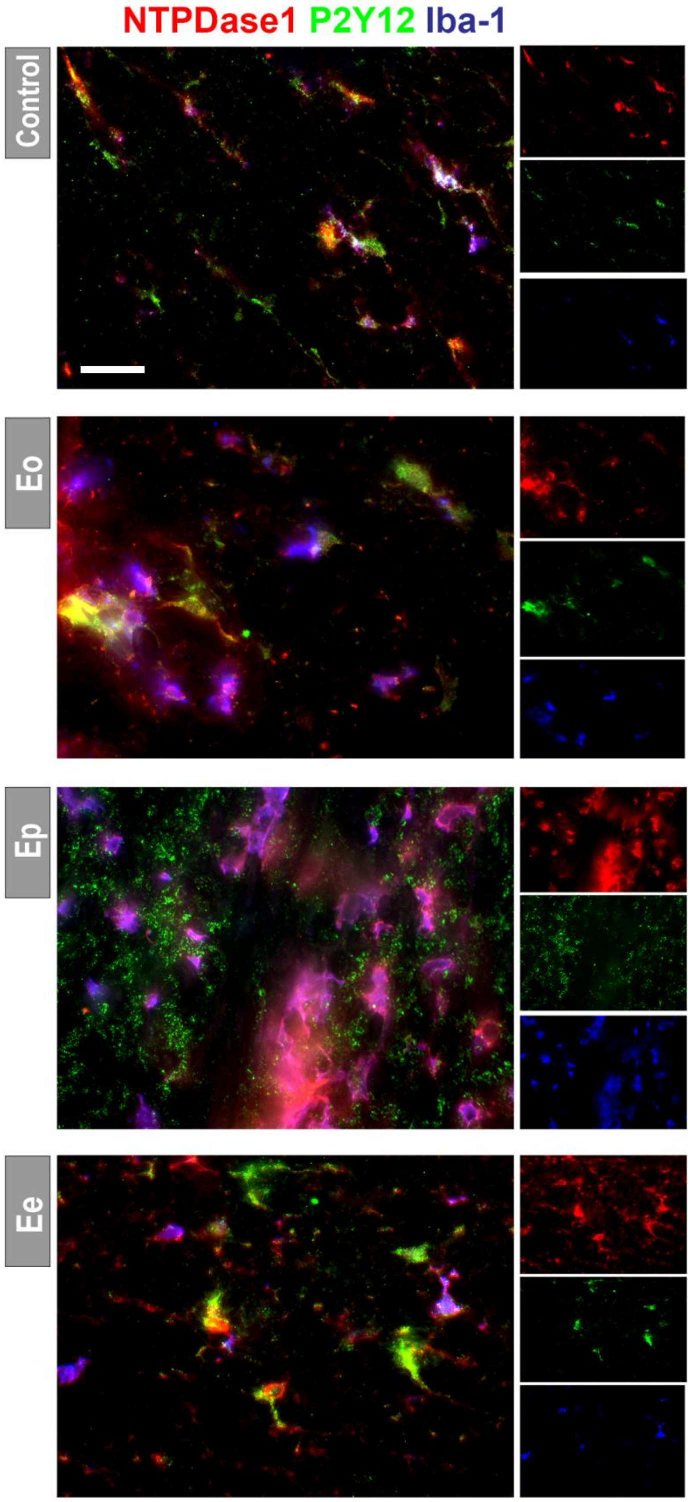

For reviewer Fig. 1. Expression of $P2Y_{12}$ receptor during the course of EAE. Representative micrographs showing triple immunofluorescence labeling directed to NTPDase1 (*red fluorescence*), Iba1 (*blue fluorescence*) and $P2Y_{12}$ (*green fluorescence*) at spinal cord cross-

sections obtained from control animals and during EAE. Scale bar applicable to all micrographs = 20 μm.
